# Supplementary material for: Design of complicated all-α protein structures
Source: Nat Struct Mol Biol. 2024 Jan 4;31(2):275–82. doi: 10.1038/s41594-023-01147-9 (PMC11377298; doi:10.1038/s41594-023-01147-9)
Supplement: Supplementary file 1 — Supplementary Text, Figs. 1–12 and Tables 1–9. [file 41594_2023_1147_MOESM1_ESM.pdf]

nature structural & molecular biology

Article https://doi.org/10.1038/s41594-023-01147-9

Design of complicated all-α protein structures

In the format provided by the authors and unedited

Table of Contents

Supplementary Text 2

Supplementary Fig. 1 | Backbone structures of the five designed topologies. 9

Supplementary Fig. 2 | HSQC for the 23 designed proteins. 10

Supplementary Fig. 3 | Results of SEC-MALS. 12

Supplementary Fig. 4 | 2D ^{1}H-^{15}N HSQC spectra of experimentally determined structures. 18

Supplementary Fig. 5 | Solved structures. 19

Supplementary Fig. 6 | Energy landscapes for the designs with Ala mutations for hydrophobic or helix-capping residues. 21

Supplementary Fig. 7 | The number of distance constraints for each residue obtained for the NMR structure determination 22

Supplementary Fig. 8 | A loose packing of the C-terminal helix of H6_fold-U_Nomur 23

Supplementary Fig. 9 | Experiments for ^{15}N-{^{1}H} NOE, ^{15}N relaxation rates (R2/R1), and 2D ^{1}H-^{15}N CLEANEX-PM FHSQC for H6_fold-U_Nomur. 24

Supplementary Fig. 11 | Distribution of radius of gyration for naturally occurring all-α proteins 27

Supplementary Fig. 12 | Thermal denaturation of Elsa with the C-terminal His-tag. 28

Supplementary Table 1 | Designed sequences and sequence identity among them. 34

Supplementary Table 2 | Summary of experimental results of 10 designs for H5_fold-0 35

Supplementary Table 3 | Summary of experimental results of 7 designs for H6_fold-C 36

Supplementary Table 4 | Summary of experimental results of 7 designs for H6_fold-Z. 37

Supplementary Table 5 | Summary of experimental results of 8 designs for H6_fold-U 38

Supplementary Table 6 | Summary of experimental results of 8 designs for H7_fold-K 39

Supplementary Table 7 | Completeness of assigned atoms in NMR structure determination 40

Supplementary Table 8 | Summary of experimental results for the six designs of the five folds. 41

Supplementary Table 9 | Comparison of ABEGO-based loop geometries of HLH motifs between design models and experimental structures for the six designs of the five folds 42

Supplementary Text

Detailed description on designed folds

H5_fold-0

H5_fold-0 is a 5-helix structure (H.O. = 0.22) with 90 residues, of which the backbone structure topology was found as the one with the smallest values for the radius of gyration and H.O. in the combinatorically generated structures. The overall structure shows a snail-like shape with a right-handed screwed α-helices arrangement. This fold comprises four HLH motifs. The first one is the v-shaped type motif (the loop ABEGO pattern is BAABB), similar to the first loop in protein A (PDB code: 1BDD), making the connected helices form a left-handed packing. The second and fourth ones are the corner-type motifs (GBB), identical to one found at the DNA binding site in homeodomains; the loops in the two motifs were found to have different sequences after the Rosetta sequence design, due to different structural environments: GRS and GIT for the second and fourth loops, respectively. The third loop is composed of a residue with the backbone torsion E in the ABEGO representation, making the flanking α-helices v-shaped. The two anti-parallelly aligned α-helices (the first and second helices, and the third and fifth helices) make the space for the protein core, and the shortest fourth helix with eight residues, not making any helix-helix pairings, covers the core space as a lid.

H6_fold-C

H6_fold-C is a 6-helix structure (H.O. = 0.31) with 112 residues. This fold can be divided into two halves, each consisting of anti-parallelly aligned three helices: one is the second, third, and fourth helices, and the other is the first, fifth, and sixth helices. These two halves are arranged crosswise and the core space is formed between the two. This fold comprises five HLH motifs; the first one is the corner-type motif (the loop ABEGO pattern is B); the second one is the hairpin-type motif (GBB) with a left-handed helix-helix packing; the third one is the hairpin-type motif (GB) with a right-handed helix-helix packing; the fourth one is the corner-type motif (B) with a wider bending angle than the first one; the fifth one is the hairpin-type motif (BAAB) with a right-handed helix packing.

H6_fold-Z

H6_fold-Z is a 6-helix structure (H.O. = 0.24) with 121 residues. The five HLH motifs are composed of three corner, one v-shaped, and one hairpin types. This fold shows the Z-like arrangement of the first three helices and the triangle arrangement of three helices from the second to fourth helices. Since the structure contains only one hairpin-type motif, most of the helix-helix packing interactions largely deviate from canonical ones. The fourth HLH motif includes the short GB loop and have a right-handed packing form. The fifth one is the corner type motif (GBB), in which the loop is relatively buried by the third helix, thus one of the hydrogen-bond donors in the loop, the oxygen atom of Gly99, is satisfied through the hydrogen bonding with the Gln49 in the third helix. In addition, this 90° corner bending places the sixth helix in between the first and second helices. The first and sixth helices form a non-local parallel helix-helix packing. The overall topology is complicated and has the arrangement slightly similar to the globin fold.

H6_fold-U

H6_fold-U is a 6-helix structure (H.O. = 0.48) with 113 residues. This fold has an apparent structural similarity to H6_fold-C. This fold also can be divided into two halves, each consisting of anti-parallelly aligned three helices: one is the third, fourth, and fifth helices, and the other is the first, second, and sixth helices. These two halves are arranged almost crosswise and the core space is formed between the two. This fold comprises five HLH motifs; the first one is the hairpin-type motif (the loop ABEGO pattern is BAB); the second one is the v-shaped-type motif (BB); the third one is the hairpin-type motif (AGB); the fourth one is the v-shaped-type motif (AGBB); the fifth one is the corner-type motif (AG). The last HLH motif showed an AGEB loop geometry in the NMR structure, although the overall arrangement of α-helices agreed well. This fold has nonlocal contact patterns similar to Greek-key motifs; non-adjacent secondary structure pairs have many nonlocal contacts.

H7_fold-K

H7_fold-K is a 7 helix structure (H.O. = 0.50) with 128 residues. Among the designed folds, this fold shows the highest H.O. value and bundle-like structure. This fold comprises six HLH motifs. The first HLH motif with a single residue loop (the ABEGO pattern is A) forms a kinked helix structure, which is stabilized by optimized hydrophobic packing around it. The second one

is the hairpin-type motif (the loop ABEGO pattern is AGABB); the third one is the hairpin-type motif (AGB); the fourth one is the v-shaped-type motif (AGBB); the fifth one is the v-shaped-type motif (BAB); the sixth one is the corner-type motif (AB). This fold can be divided into two halves: one is the first to the fourth helices and the other is the fifth to the seventh helices. A large hydrophobic core is formed between these two halves, which may explain the highest denaturation temperature among all the five designed folds.

NMR structure determination

Samples for all designed proteins were stable during NMR structure determination (for more than 2-3 weeks) at the concentration (0.5-1.0 mM), and rarely minor components were found. No noticeable signal changes in 2D ^{1}H-^{15}N HSQC at different concentrations (2-10 times dilution) were identified.

Structural determination was performed by perfectly blind analysis to avoid any biases being involved in the automated NMR analysis: the analyst never has known the designed structures even their sequences before structure determination. The NMR spectrometers at the ^{1}H resonance frequencies of 700 and 800 MHz equipped with the 2nd or 3rd generation Cryo-probes for high sensitivity (Bruker) and highly concentrated samples yielded a significant number of NOE peaks from NOESY type spectra for all samples as shown in the following supplementary tables. For each sample, around 90% of the NOE peaks were assigned by CYANA and the well-converged structure was obtained, supporting high consensus between the NOE peaks and the calculated NMR structure.

RDC values were exclusively used for assessment of the determined NMR structures. This strategy enhances the reliability of the determined NMR structures after refinement calculations by Amber12, which have the geometrical normality (such as Ramachandran plots and vdW clash) and low violations of restraints. RDC can be affected by local motions of HN-N vectors in a wide range of time scale, but the effect is not large if a sufficient number (the coverage of residues more than 80%) and sufficient amplitude of RDC values (> ±10 Hz with error < 0.2 Hz) are obtained. Moreover, the α-helices in the designed structures were designed to have largely different orientations, thus this RDC analysis using $^{1}D_{1H\text{-}15N}$ is suitable for structure validation.

H5_fold-0_Chantal

One of the structural features in the design and determined NMR structures for H5_fold-0_Chantal is the tightly stacked aromatic rings between His63 and Tyr59. The imidazole ring of His63 was assumed to be protonated on Nε2 based on the chemical shift of His63-^{13}Cδ1. Therefore, Nε2 of His63 was protonated in the CYANA and Amber12 calculations. As shown in 2D ^{1}H-^{15}N HSQC in Supplementary Fig. 4, H5_fold-0_Chantal does not show any minor components at the NMR condition. The signal of Arg22-^{1}Hε/^{15}Nε is up-field shifted by ring-current effect of Trp33, strongly indicating interactions between side-chains of Arg22 and Trp33. The His63-Hδ2/Cδ2 signal in 2D ^{1}H-^{13}C HSQC supported the tightly packed aromatic rings between His63 and Tyr59. Several methyl and methylene signals are also up-field shifted for Ile74-Hγ2, Val11-Hγ1/2, Leu56-Hγ1/2, Arg22-Hγ2/3 and Arg22-Hβ2/3. These atoms are close to the aromatic rings of Phe14, Tyr18, Trp22, and Tyr59, forming tightly packed hydrophobic core. The structure validation by PALES using almost all of the RDC values (82 out of 88, excluding flexible regions) exhibits the high correlation coefficient, 0.948 (this is higher than designed structure (correlation coefficient = 0.894)), indicating that the NMR structure was accurately determined.

<u>H6_fold-C_Rei</u>

The side-chain orientations of the core, Ile, Val, Leu, Met, Phe, and Trp, in the designed structure show remarkable similarity with those of the NMR structure. On the other hand, the side-chain orientation of Trp61 is slightly different between the NMR and designed structures, suggesting that the local packing around the side-chain may not be sufficient enough to fix its conformation. Interestingly, the methyl group of Leu12 located in the position buried by Trp61 does not seem to be shielded. The fluctuation of the Trp61 side-chain likely impairs the packing between Trp61 and Leu12. Only Val76-Hγ1/2, Leu25-Hδ1/2 methyl protons were found to be shielded by Phe82. A lot of methyl groups are considered to contribute the tightly packed hydrophobic core. Interestingly, Gln36 -Hε21 and -Hε22 signals are unusually shielded and down-field shifted. This effect is caused by strong hydrophilic interactions with the side-chains of Gln84 and Glu32, respectively, forming hydrogen bonds to stabilize the helical structure. The side-chain of Asn96 was found to make the N-terminal capping for the following α-helix. The correlation coefficient of RDC validation score for the determined NMR structure using 102 of

107 RDC values (-24.2~19.83 Hz) except for flexible regions was 0.923 (this is slightly higher than that for the designed protein, correlation coefficient = 0.916).

<u>H6_fold-Z_Gogy</u>

This protein has a characteristic Met-cluster comprised of the residues 13, 16, 20, 76 and 112. In the NMR structure, the cluster forming a small pocket tightly accommodates the side-chain of Trp73, while in the designed structure, the indole of Trp73 is slightly away from the cluster. Methyl signals of Val72-Hγ1/2, Leu98-Hδ1/2 were up-field shifted by Phe94. Phe94 and Phe108 are packed, supported by the strongly shielded Phe94-Hζ signal by the ring current of the Phe108 side-chain. Although a lot of signals are overlapped in 2D ^{1}H-^{15}N IPAP-HSQC, $^{1}D_{1H-15N}$ values (-23.60~30.78 Hz), the signals for 95 of 116 residues, covering all α-helices, were obtained. The correlation coefficient calculated by PALES for the determined structure was 0.913, while that of designed structure was slightly better, 0.921, indicating that both the NMR and designed structures well satisfy experimental data.

<u>H6_fold-U_Nomur</u>

Many amide signal intensities were reduced and some signals were missing because of the buffer condition with higher pH and salt concentration, and some signals were missing. In addition, many amide and methyl signals were crowded in narrow regions because of the high helix content in the structure as well as many Ala residues in the design sequence. The automated assignments for H6_fold-U_Nomur was first performed by FLYA with additional spectra such as 3D HN(CA)CO, HNCA, HN(CO)CA, (H)CC(CO)NH and H(CCCO)NH. The sequence specific assignments were confirmed and corrected by 3D H(CA)NNH, (H)N(CA)NH, HBHA(CO)NH, HNHAHB spectra. The assignments of side-chain signals were also confirmed and corrected by 3D ^{13}C-HSQC (^{13}C-time domain) NOESY ^{13}C-HSQC, ^{13}C-HSQC (^{13}C-time domain) NOESY ^{15}N-HSQC and 4D 13C-HSQC NOESY 13C-HSQC spectra. For obtaining RDC values, severely overlapped signals in 2D IPAP ^{1}H-^{15}N HSQC spectra, 77 of $^{1}D_{1HN-15N}$ and 64 of $^{1}D_{1H\alpha-13C\alpha}$ were derived from 3D HN(CO)CA-J measured at the same alignment condition. Each value in the RDC table was applied to the PALES calculation, and 141 values were used for the final calculation. The N- and C-terminal helices tend to show larger error in the RDC simulation, indicating that these helices may not be well packed. Despite the low correlation

coefficient (0.776) for the designed protein, the overall designed structure should be the same as the NMR structure. On the other hand, the aromatic ring positions of Phe51, Phe54 and Trp43 in the NMR structure were largely different from those in the designed structure. Leu35-Hδ1/2, Leu90-Hδ1/2 are shielded by the ring current, obviously derived from Trp43 and Phe54, respectively. These aromatic rings in the designed structure may not be able to shield any methyl groups of Ile, Val and Leu. These support that the NMR structure is more trustful than the designed structure.

H7_fold-K_Mussoc

Many amide signals were weak or disappeared in ^{1}H-^{15}N HSQC, because of the higher pH and salt concentration, and many signals were crowded because of the high helix-content in the designed structure. After the automated signal assignments by FLYA, sequence specific assignments were confirmed by 3D (H)N(CA)NH and ^{13}C-HSQC (^{13}C-time domain) NOESY ^{15}N-HSQC spectra. Side-chain signal assignments for aliphatic residues were corrected by 3D (H)CC(CO)NH, H(CCCO)NH, and (H)CCH-TOCSY. Several proximities of methyl groups were successfully confirmed on 4D NOE spectra to make sure how correctly assigned crowded methyl signals and related NOEs were. The methyl of Leu71-Hδ1/2 are strongly shielded by Phe23 and the methyl group, Ile41-Hγ2, Leu103-Hδ1/2, and Leu113-Hδ1/2, are strongly shielded by Trp109. Trp109-Hζ3 is strongly shielded by the ring current effect of the Phe63 side-chain, indicating the hydrophobic side-chains are correctly packed in the NMR structure.

Supplementary Figures

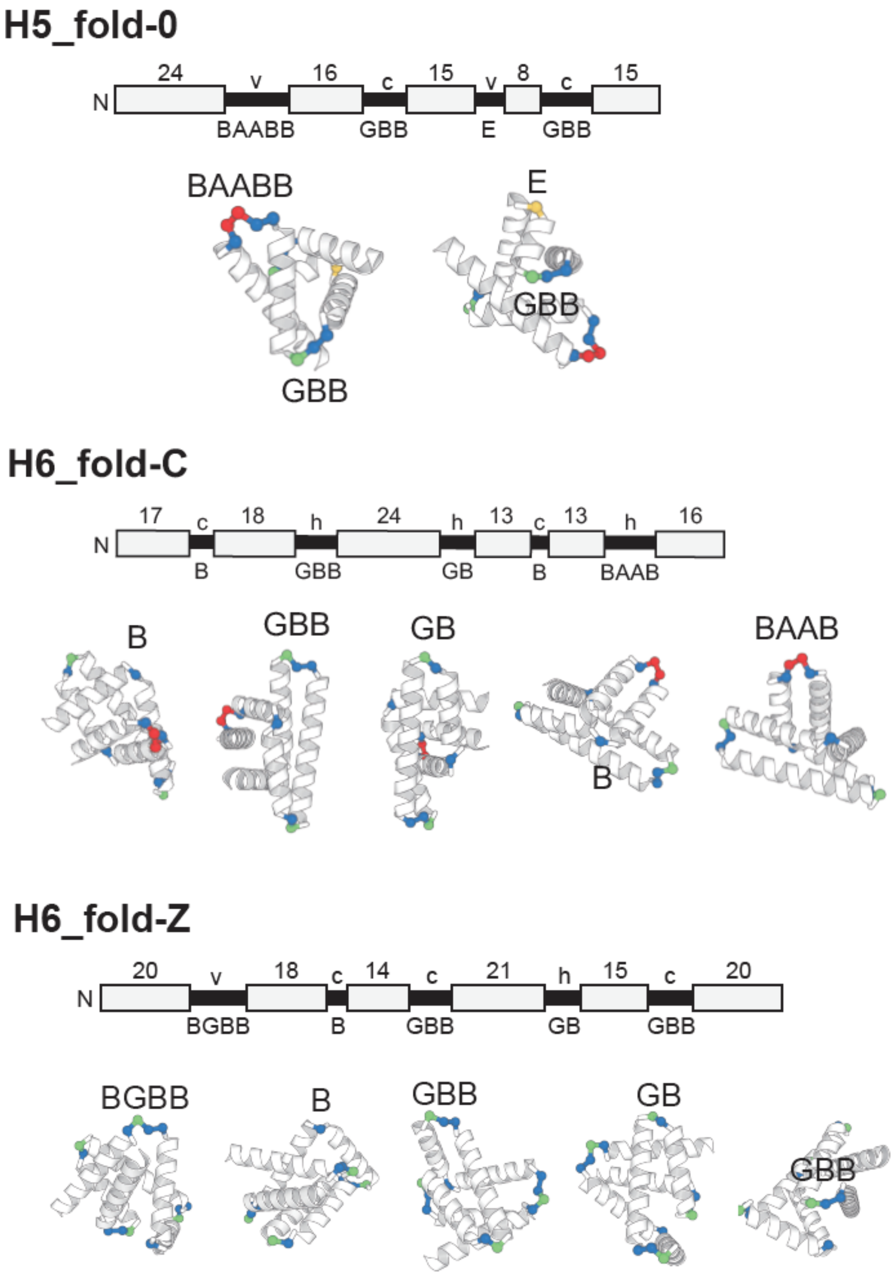

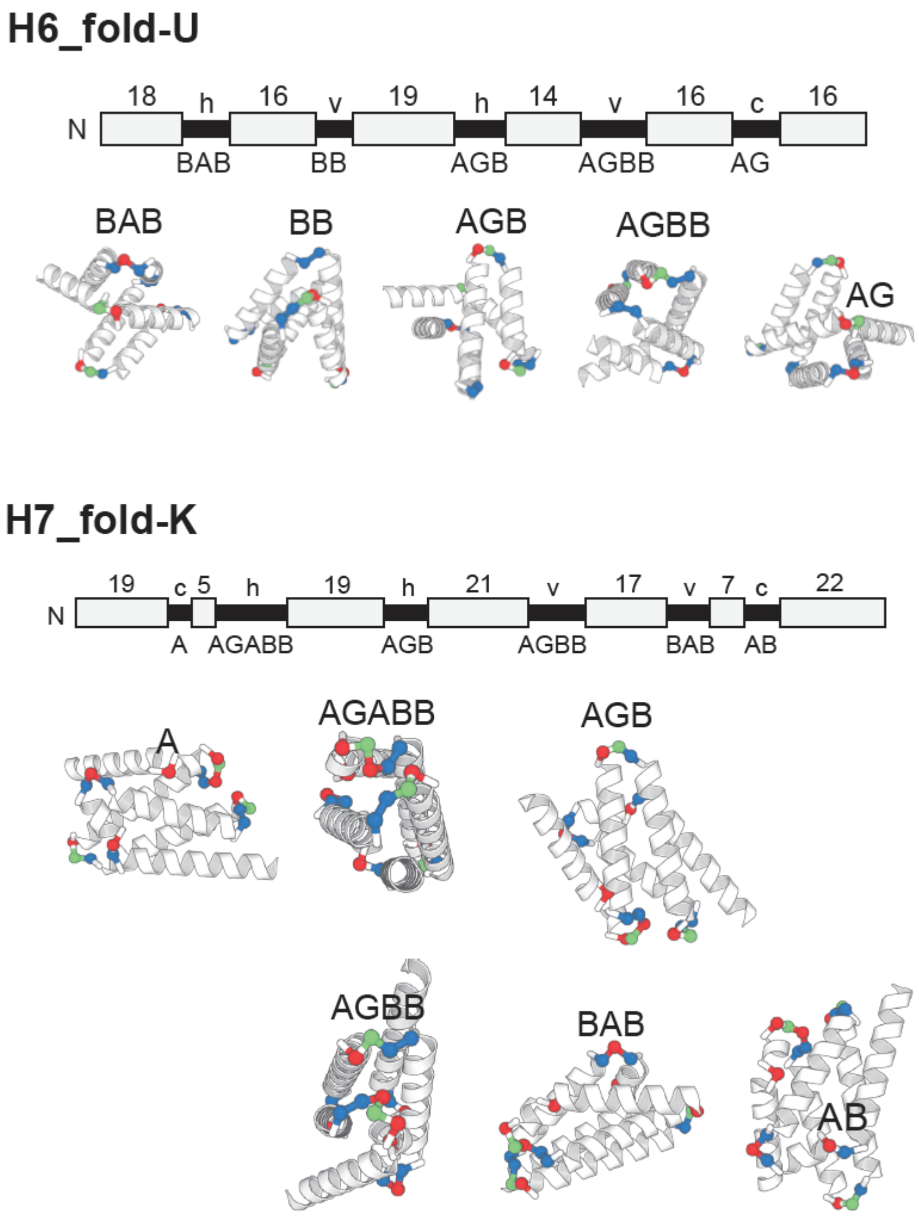

Supplementary Fig. 1 | Backbone structures of the five designed topologies.

Backbone structures with different views to show each loop and its surroundings. The residues with the backbone torsion angle, A, B, E, and G, in the ABEGO representation, are shown in red, blue, yellow, and green, respectively.

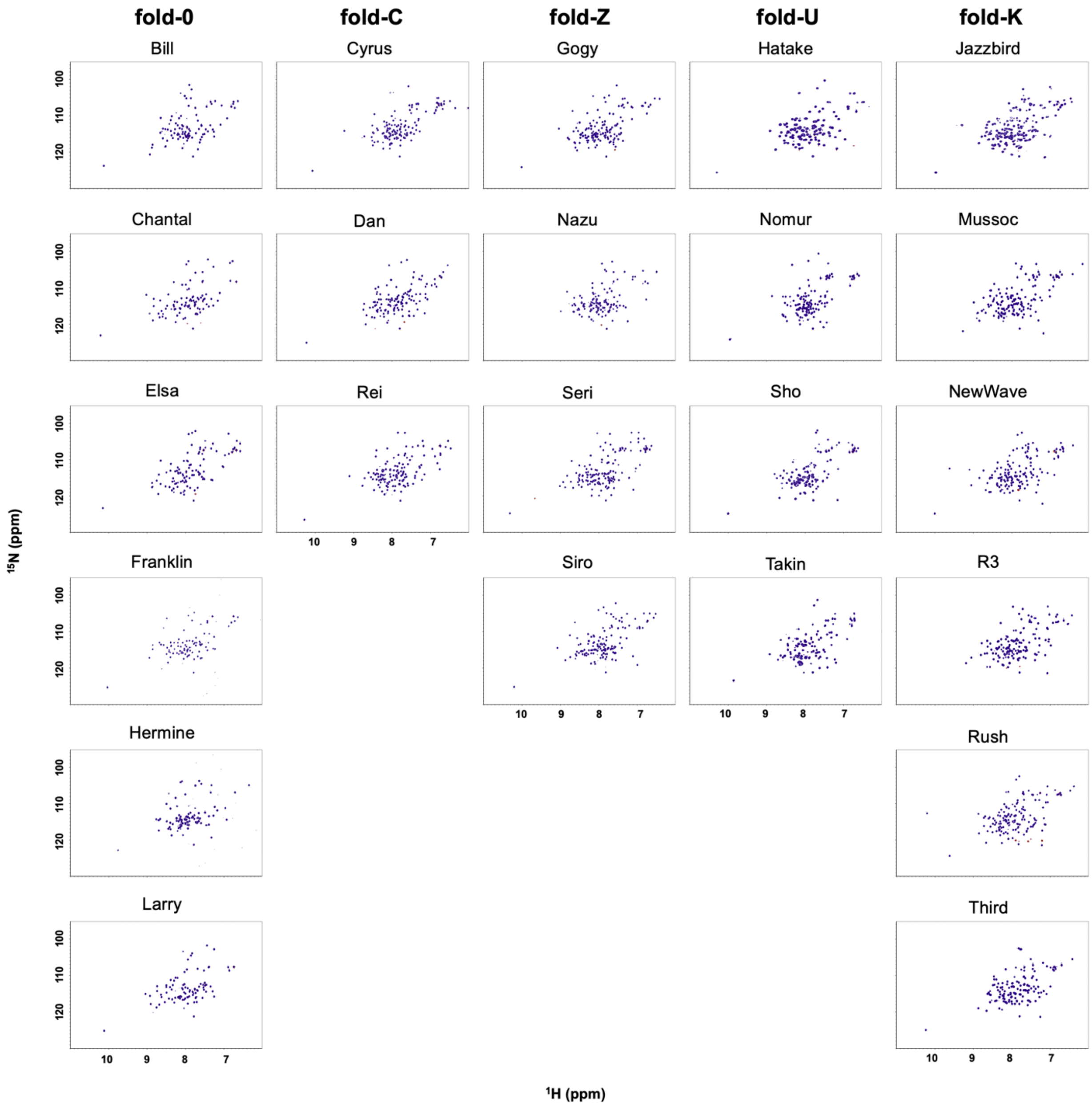

Supplementary Fig. 2 | HSQC for the 23 designed proteins.

Two-dimensional ^{1}H-^{15}N HSQC spectra at 25 °C and 600 MHz are shown for the 23 designed proteins. All spectra show well-dispersed sharp peaks typically observed for helical proteins.

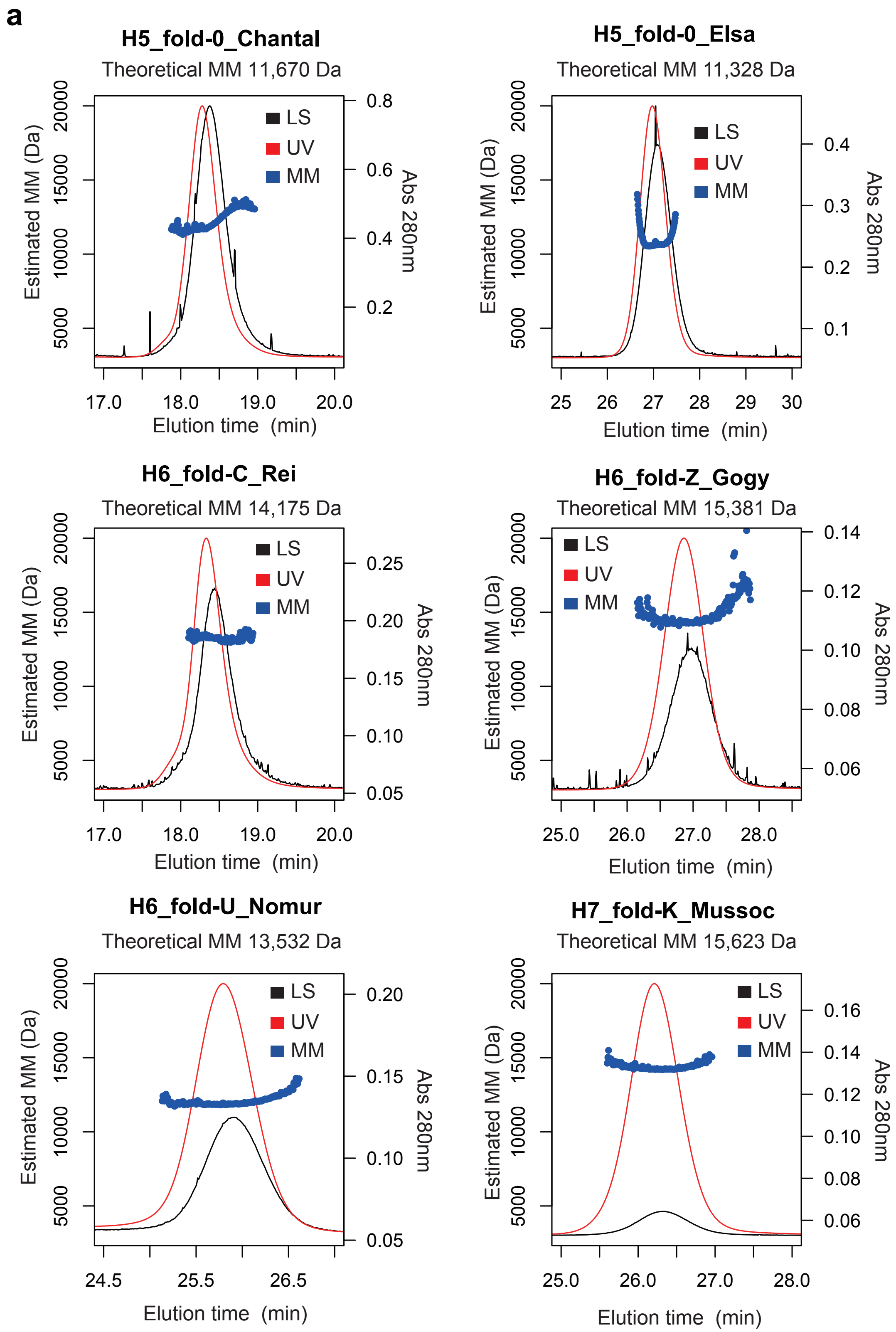
a
H5_fold-0_Chantal
Theoretical MM 11,670 Da
H5_fold-0_Elsa
Theoretical MM 11,328 Da
H6_fold-C_Rei
Theoretical MM 14,175 Da
H6_fold-Z_Gogy
Theoretical MM 15,381 Da
H6_fold-U_Nomur
Theoretical MM 13,532 Da
H7_fold-K_Mussoc
Theoretical MM 15,623 Da
LS
UV
MM
Estimated MM (Da)
Abs 280nm
Elution time (min)

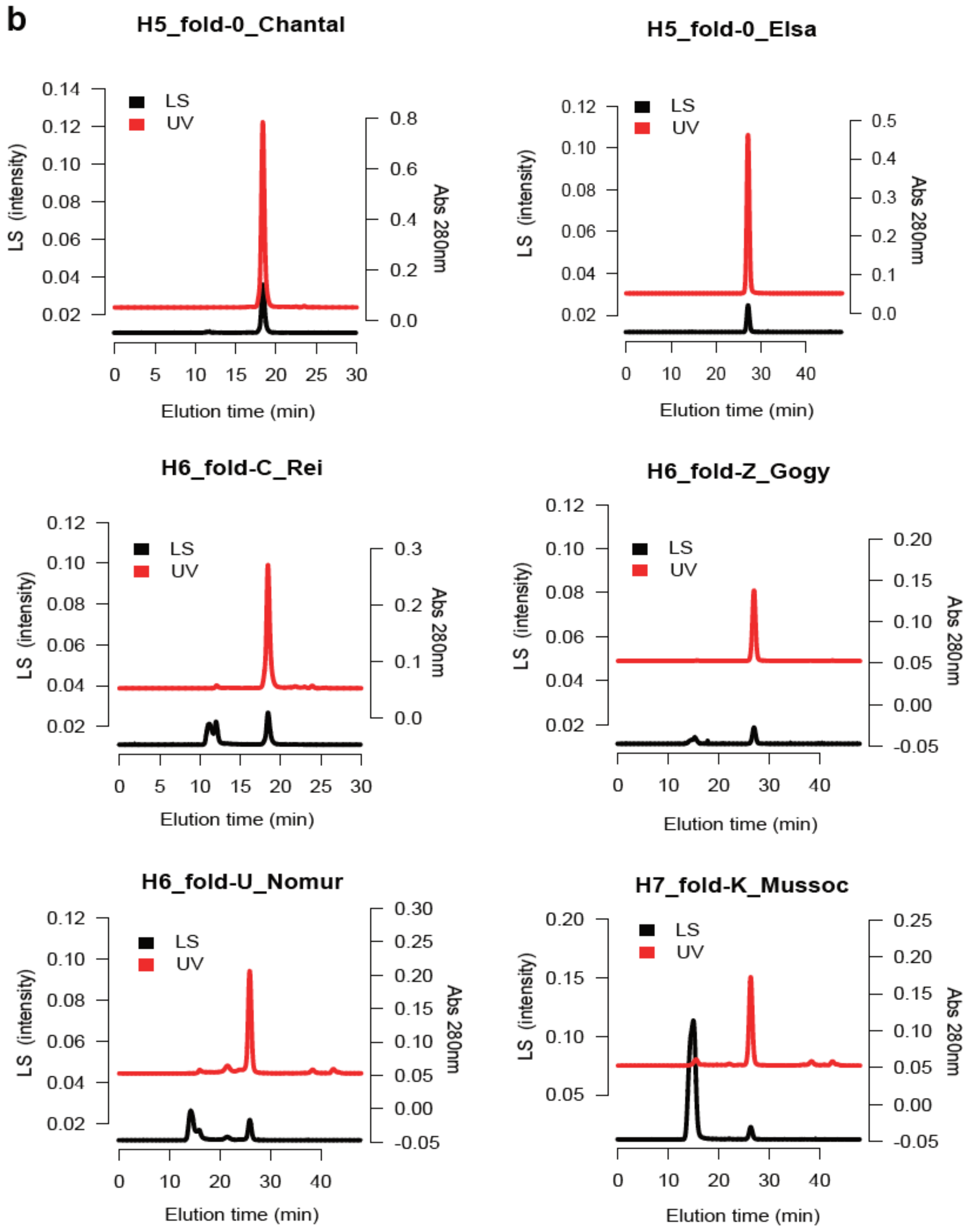

Supplementary Fig. 3 | Results of SEC-MALS.

a, Close-up views around the dominant peaks. **b**, SEC chromatograms of nickel-purified designed proteins. LS: light scattering, UV: ultraviolet, MM: molecular mass.

H5_fold-0-Chantal

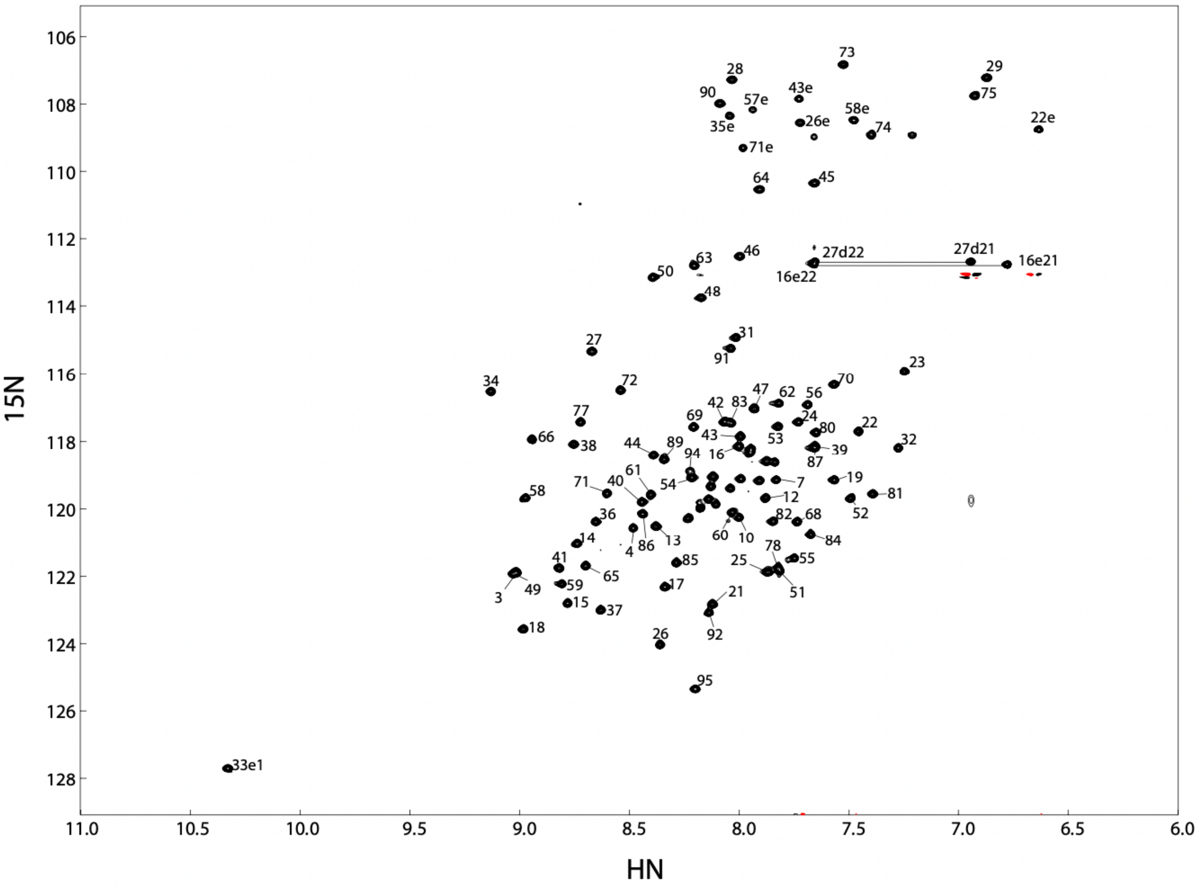

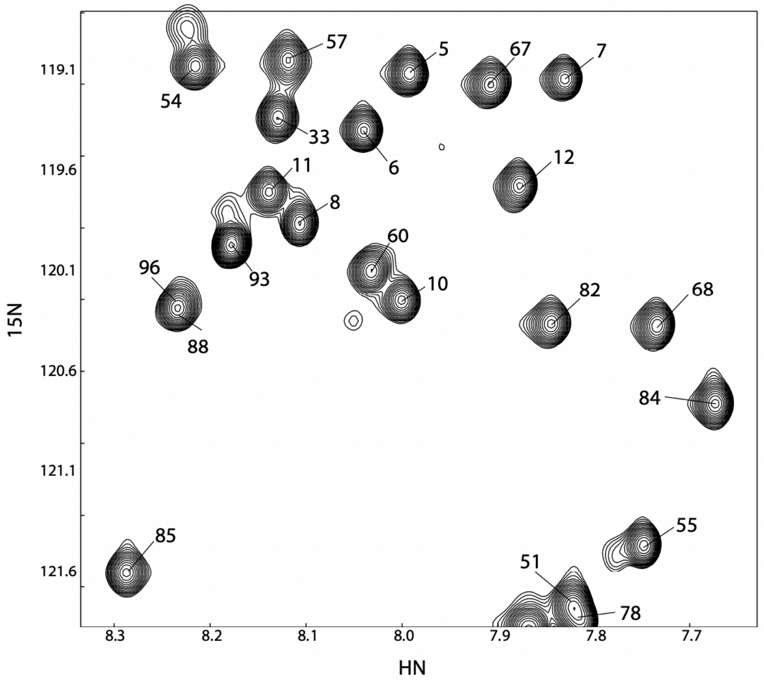

H6_fold-C_Rei

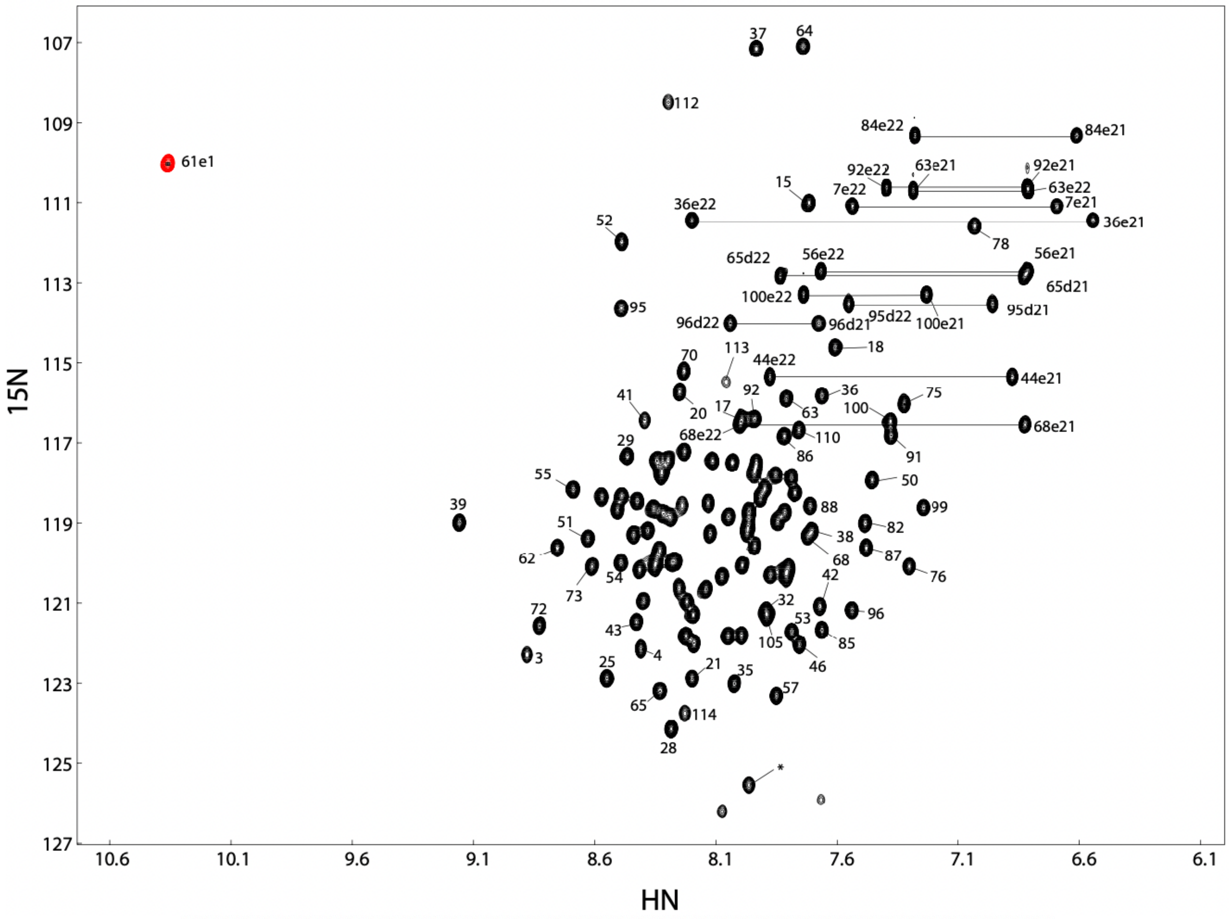
15N
HN
107
109
111
113
115
117
119
121
123
125
127
10.6
10.1
9.6
9.1
8.6
8.1
7.6
7.1
6.6
6.1

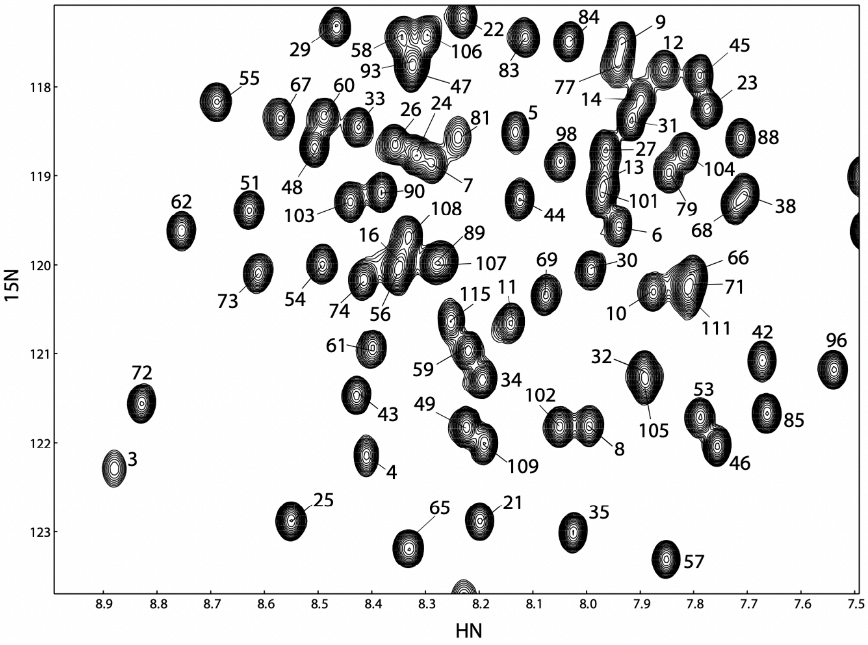
15N
HN
118
119
120
121
122
123
8.9
8.8
8.7
8.6
8.5
8.4
8.3
8.2
8.1
8.0
7.9
7.8
7.7
7.6
7.5

H6_fold-Z_Gogy

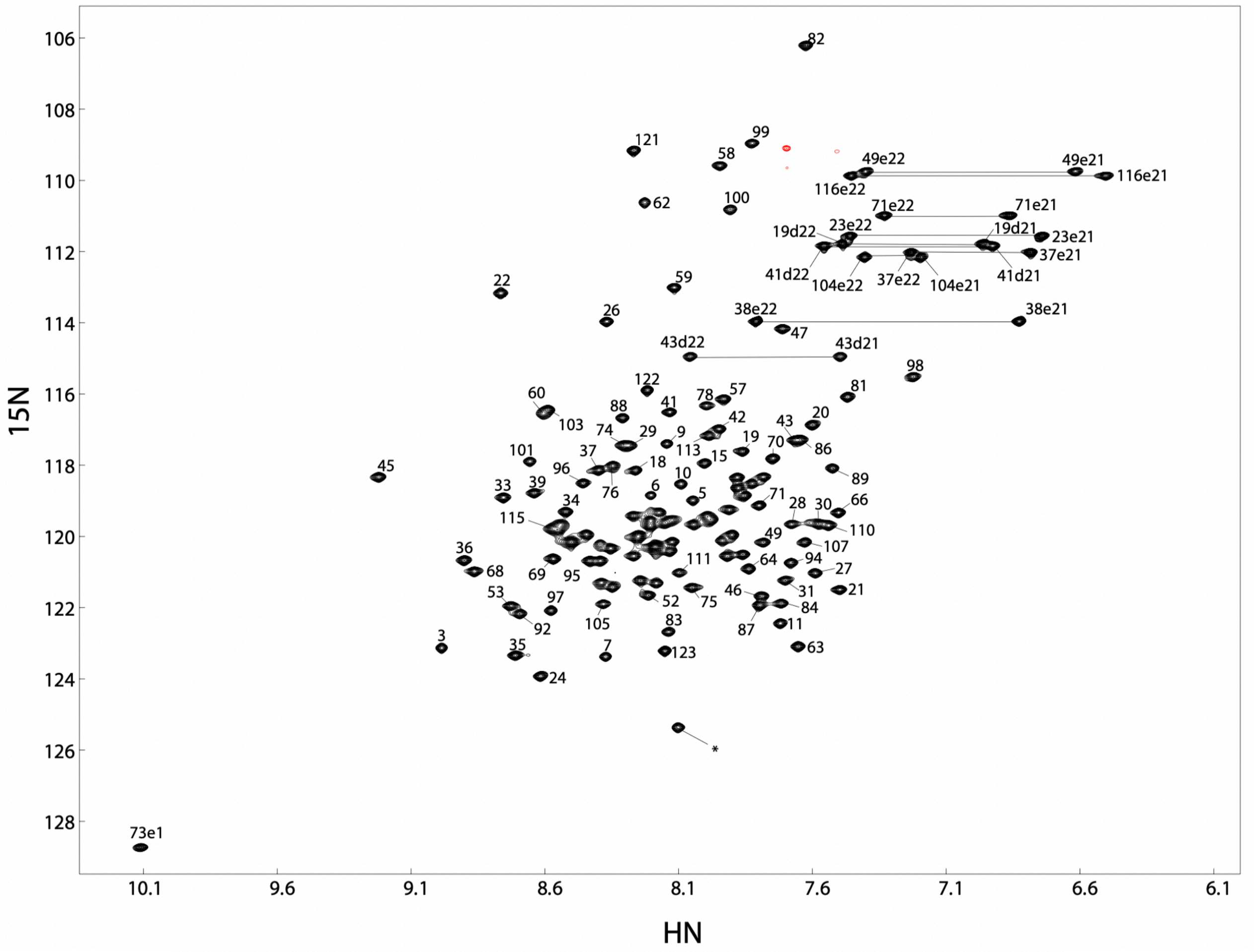

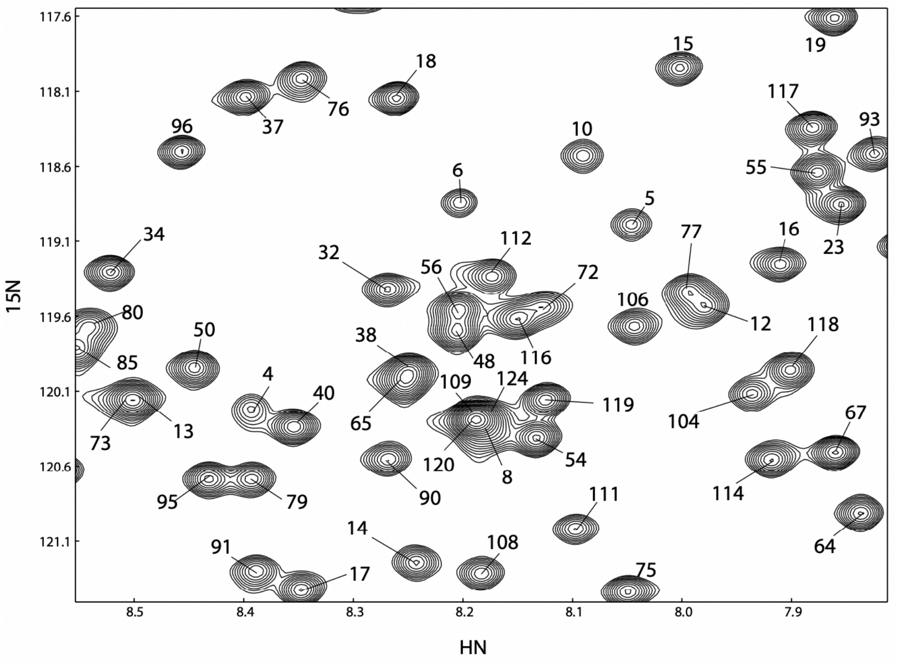

H6_fold-U_Nomur

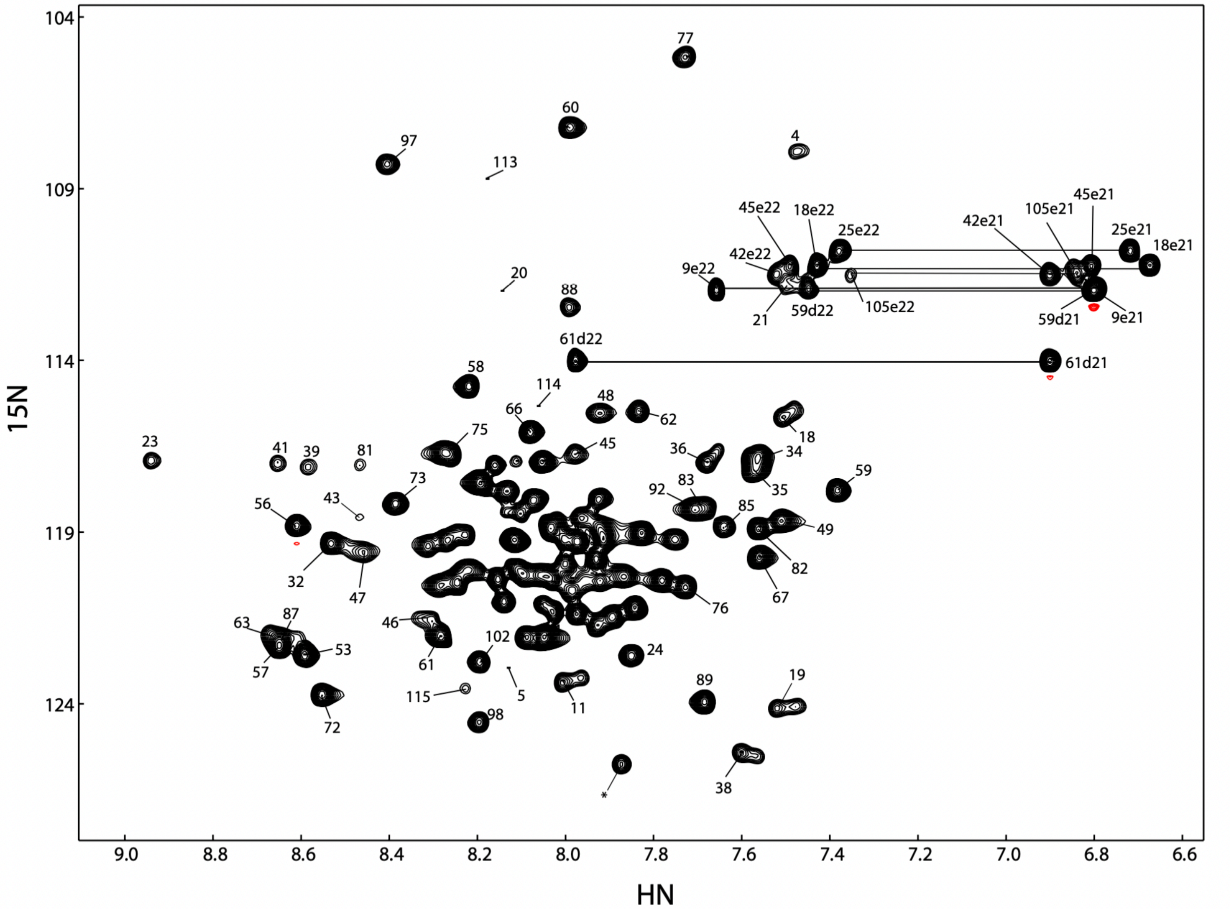

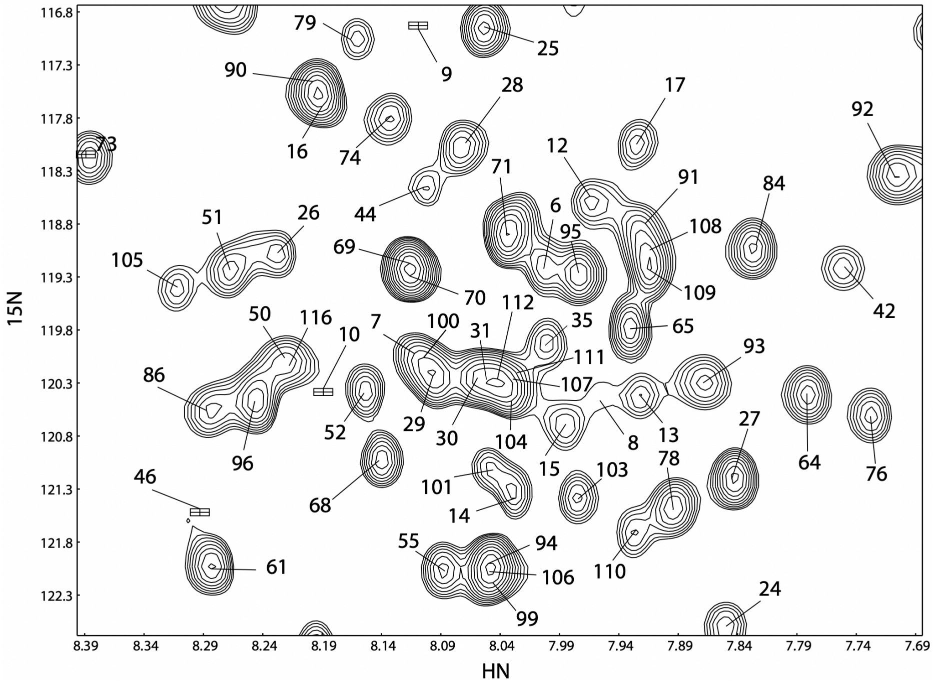

H7_fold-K_Mussoc

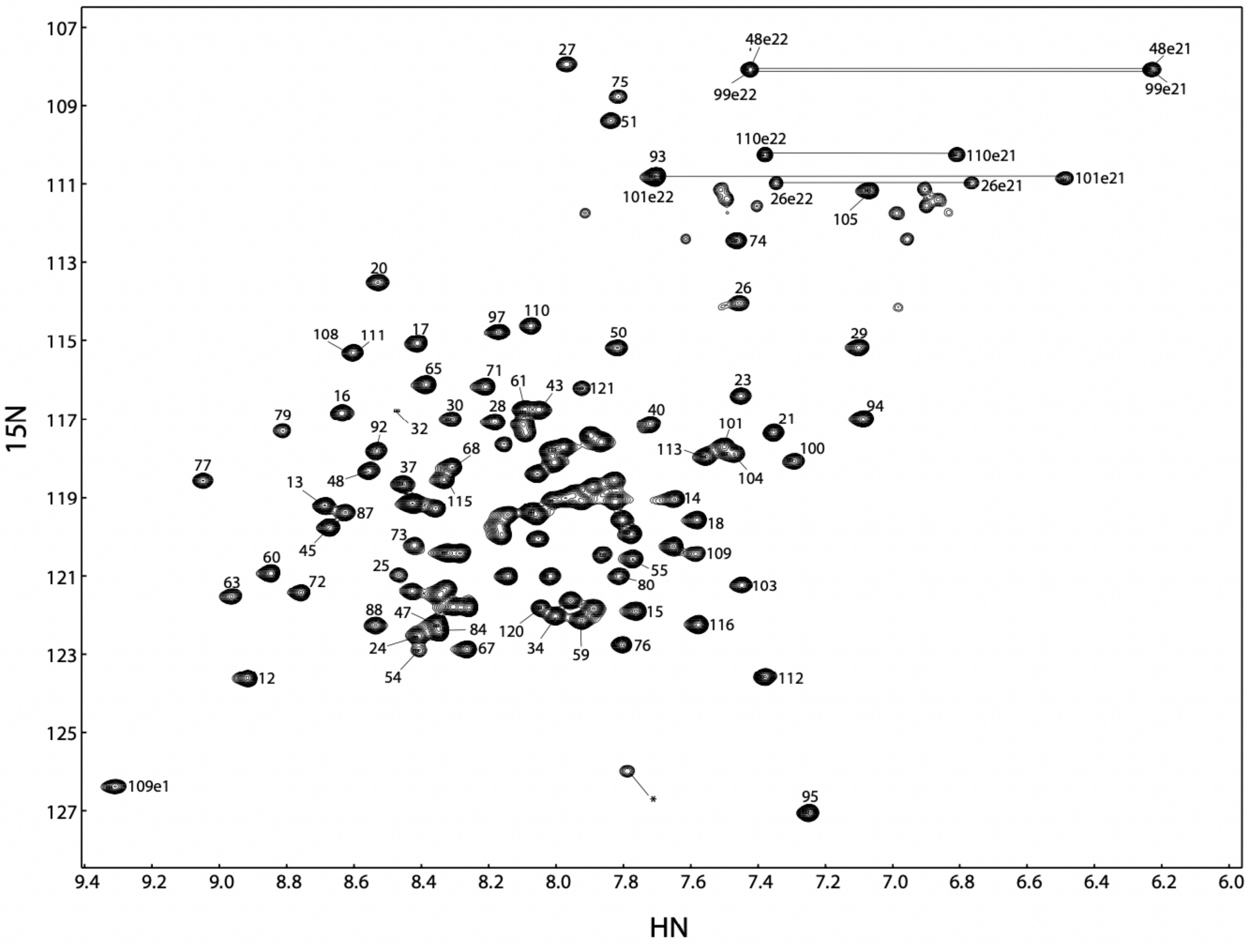

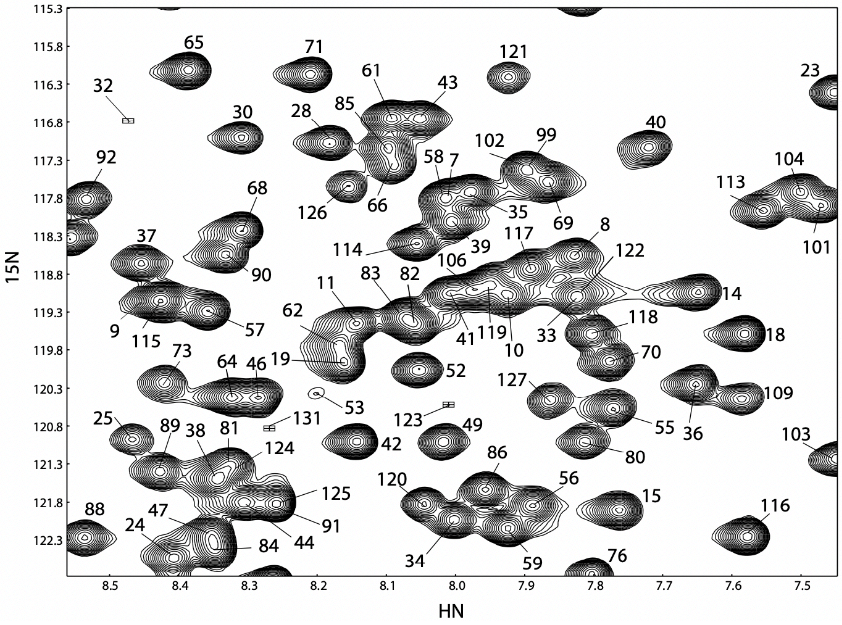

Supplementary Fig. 4 | 2D ^{1}H-^{15}N HSQC spectra of experimentally determined structures. Assigned peaks are labeled with residue numbers. For side-chain amide atoms, the labels are accompanied with d or e, representing Hδ1/Nδ and Hδ2/Nδ for Asn, and Hε1/Nε and Hε2/Nε for Gln, respectively. For Trp, e1 represents Hε1/Nε1. The lower figure in the HSQC plot for each design shows the region of the spectra where signals are severely crowded and/or overlapped. The peaks labeled with an asterisk correspond to the C-terminal residue and are determined based on their sharp line-shapes and typical chemical shifts.

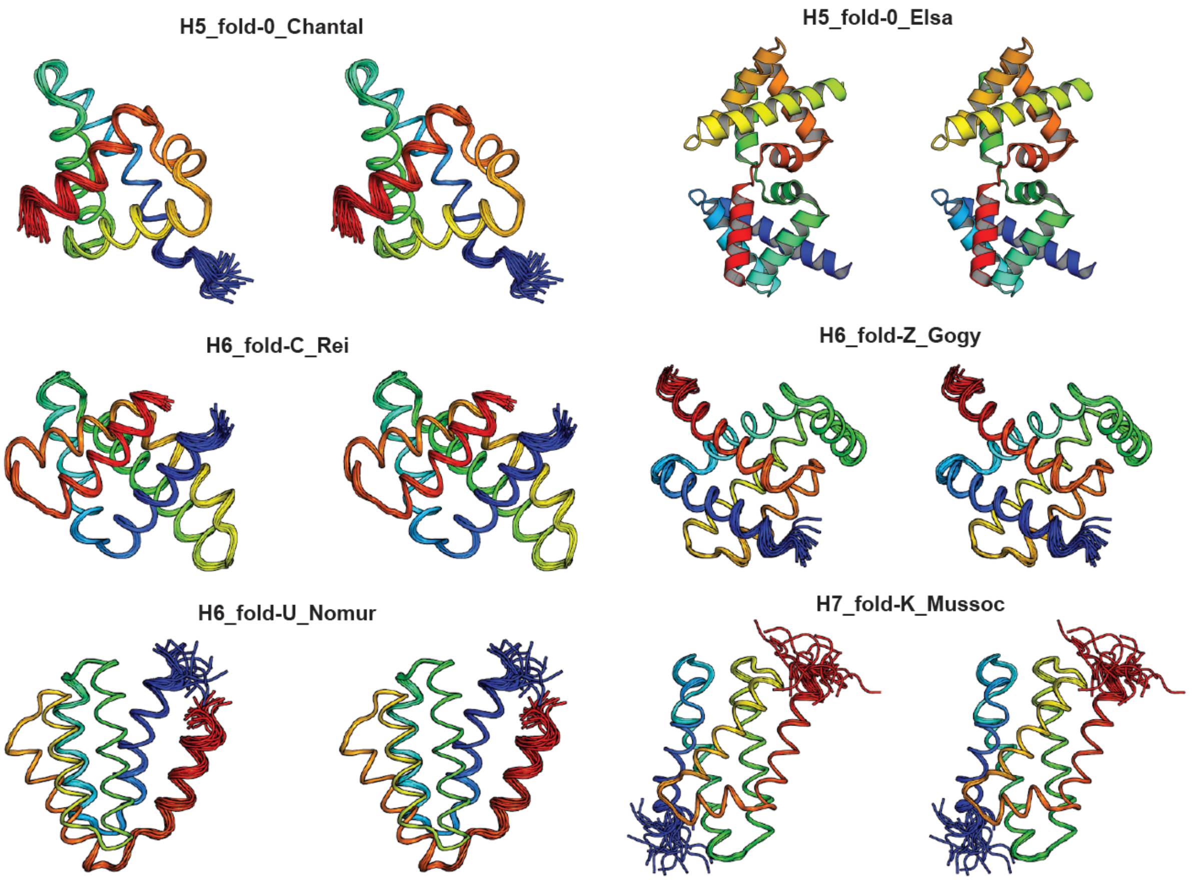

Supplementary Fig. 5 | Solved structures.

Stereo views of the solved NMR structures and the crystal structure (H5_fold-0_Elsa).

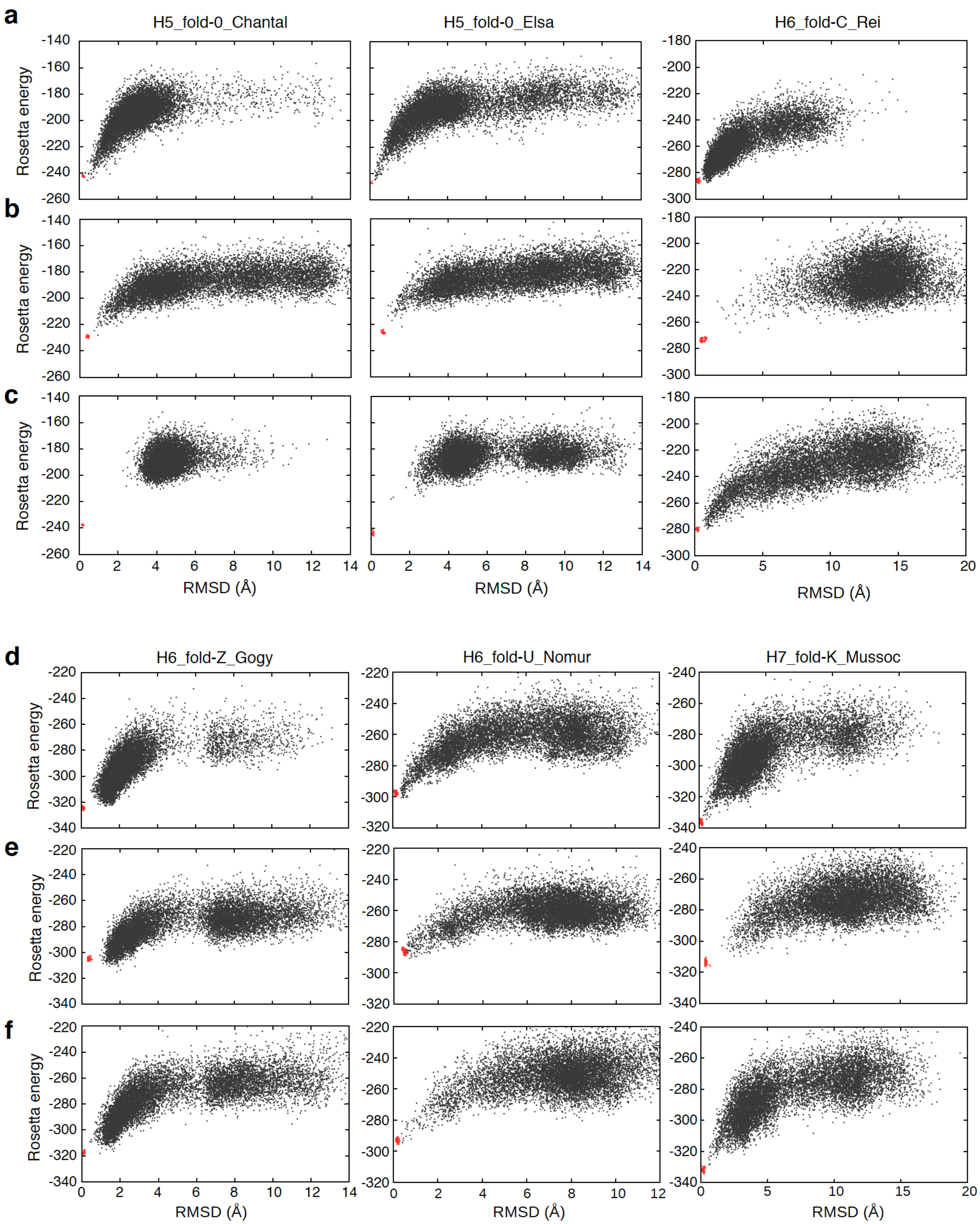

a
b
c
d
e
f
H5_fold-0_Chantal
H5_fold-0_Elsa
H6_fold-C_Rei
H6_fold-Z_Gogy
H6_fold-U_Nomur
H7_fold-K_Mussoc
Rosetta energy
RMSD (Å)

Supplementary Fig. 6 | Energy landscapes for the designs with Ala mutations for hydrophobic or helix-capping residues.

a, **d**, Energy landscapes of the original designs same as Fig. 5a. **b**, **e**, Energy landscapes for the designs with Ala mutations for hydrophobic residues in the HLH motifs. Out of hydrophobic residues within the loop and within five residues before and after the loop in the HLH motifs, the residues having hydrophobic interactions within the HLH motifs were mutated to Ala. The 10 hydrophobic residues for H5_fold-0_Chantal: W33A, V34A, V45A, V52A, V53A, Y59A, L68A, L69A, I74A, L79A; 10 residues for H5_fold-0_Elsa: W33A, V52A, V53A, Y59A, L68A, L69A, L72A, I74A, I78A, Y79A; 12 residues for H6_fold-C_Rei: L14A, V15A, L21A, L22A, L33A, I43A, W61A, V76A, L77A, F82A, M89A, V99A; 12 residues for H6_fold-Z_Gogy: M16A, M20A, V28A, M39A, V46A, L53A, V64A, I65A, F94A, L98A, I100A, V105A; 5 residues for H6_fold-U_Nomur: L35A, W43A, I82A, I83A, L93A; 12 residues for H7_fold-K_Mussoc: L16A, L20A, F23A, I29A, L34A, L56A, L71A, I81A, L92A, L100A, L103A, W109A. **c**, **f**, Energy landscapes for the designs with Ala mutations for helix capping residues in the HLH motifs. The helix-capping residues immediately before the second helix in the HLH motifs were mutated to Ala. The *y*-axis represents Rosetta all-atom energy and the *x*-axis represents the Cα root mean square deviation (RMSD) from the design model. Black points represent the lowest energy structures obtained in independent Monte Carlo structure prediction trajectories starting from an extended chain for each sequence; red points represent the lowest energy structures obtained in trajectories starting from the design model. Population shifts to unfolded structures were observed in all energy landscapes of the mutants, which indicates the importance of hydrophobic or helix capping residues in HLH motifs.

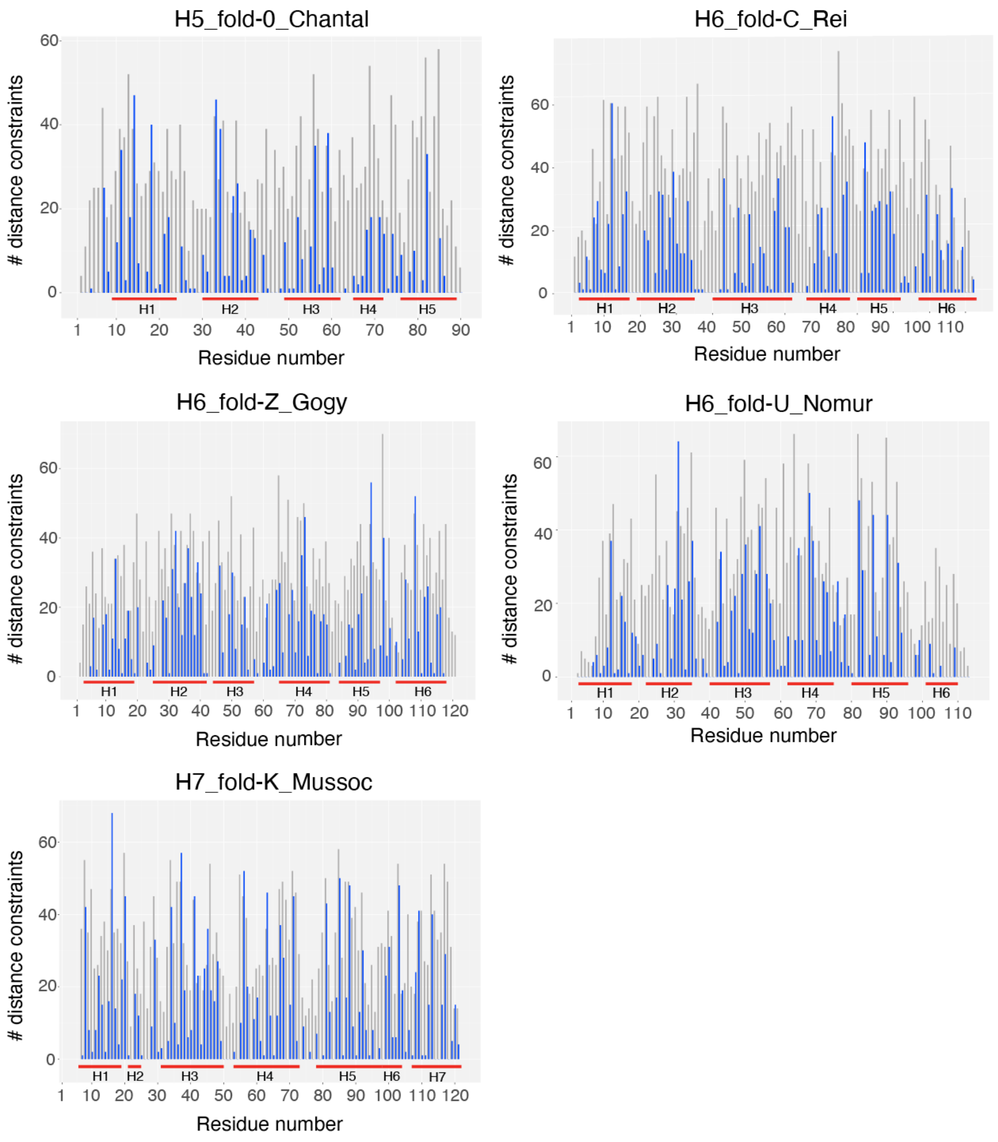

Supplementary Fig. 7 | The number of distance constraints for each residue obtained for the NMR structure determination.

Gray and blue bars respectively represent the number of local and non-local distance constraints between the i-th and j-th residues (local constraints are defined for the residues with $|i-j| < 5$, and non-local constraints are for the ones with $|i-j| \geq 5$). Red lines represent helix regions defined by the DSSP calculation for the NMR models. The number of local constraints of the C-terminal helix (H6) of H6_fold-U_Nomur indicates the helix formation. However, the number of non-local constraints for that helix is less than that of the other helices of all five designs. This result indicates that the C-terminal helix of H6_fold-U_Nomur is loosely packed despite the helix formation.

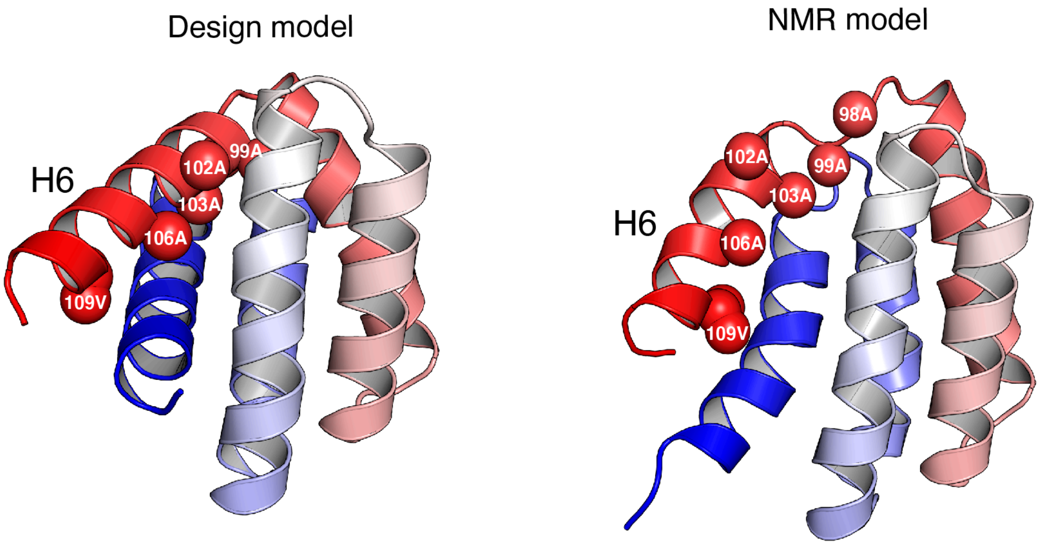

Supplementary Fig. 8 | A loose packing of the C-terminal helix of H6_fold-U_Nomur.

The design and NMR models for H6_fold-U_Nomur are shown. The sidechains of core-forming residues in the C-terminal helix are represented by spheres: except for the 109th residue, the amino acid residues are Ala.

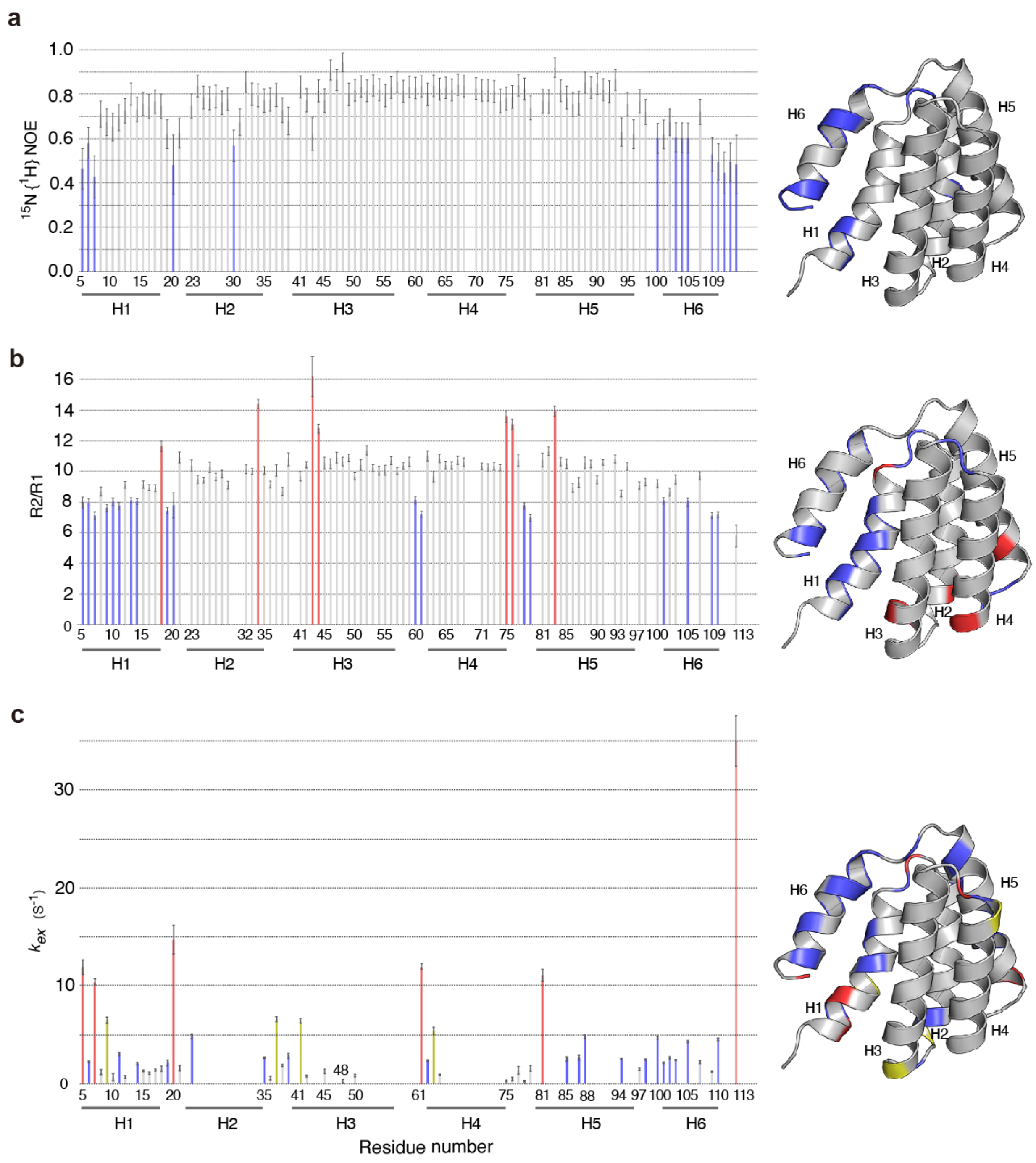

Supplementary Fig. 9 | Experiments for ^{15}N-{^{1}H} NOE, ^{15}N relaxation rates (*R2/R1*), and 2D ^{1}H-^{15}N CLEANEX-PM FHSQC for H6_fold-U_Nomur.

a, ^{15}N-{^{1}H} NOE for each residue of H6_fold-U_Nomur. The blue bars in the left figure represent data that are less than 0.6, and the corresponding residues are also colored blue in the right figure. Regions for α-helices are represented by gray lines. Residues with lower values less

than 0.6 were found in the N- and C-terminal helices as well as loops, which suggests that these regions are not unfolded but fluctuating on the psec to nsec time scale. Each error bar represents the ratio of S.D. of the noise intensities with and without a saturation pulse in the vicinity of a detected signal. The calculation follows the law of propagation of errors. **b,** The ratio of ^{15}N relaxation rates, *R2/R1*, for each residue of H6_fold-U_Nomur. The blue and red bars in the left figure represent data that are less than 8.5 and greater than 11.5, respectively, and the corresponding residues in the right figure are also colored by the same criteria. Residues with lower *R2/R1* values, shown in blue, were observed in the terminal helices, indicating their high mobility. In addition, residues with higher *R2/R1* values, shown in red, were observed, indicating the presence of chemical exchange involved in large conformational changes. Especially, the slow motion of W43 close to the terminal helices would arise from fluctuations of the terminal helices. Each error bar represents the S.D. of the residuals between measured values and the values obtained by the non-linear fitting model. **c**, The water-amide proton exchange rate for the amide group of each residue, obtained by 2D ^{1}H-^{15}N CLEANEX-PM FHSQC. The red, yellow and blue bars in the left figure represent data that are greater than 10.0 (s^{-1}), between 5 and 10 (s^{-1}), and between 2 to 5 (s^{-1}), respectively, and the corresponding residues in the right figure are also colored by the same criteria. Consistent with the ^{15}N-{^{1}H} NOE data, higher exchange rates were observed in the terminal helices as well as loops. Taken together, the N- and C- terminal helices as well as loops are fluctuating on the psec to nsec time scale, with the slow conformational exchange of W43 and nearby residues. Each error bar represents the S.D. of the residuals between measured values and the values obtained by the non-linear fitting model. See Methods for details of the above experiments.

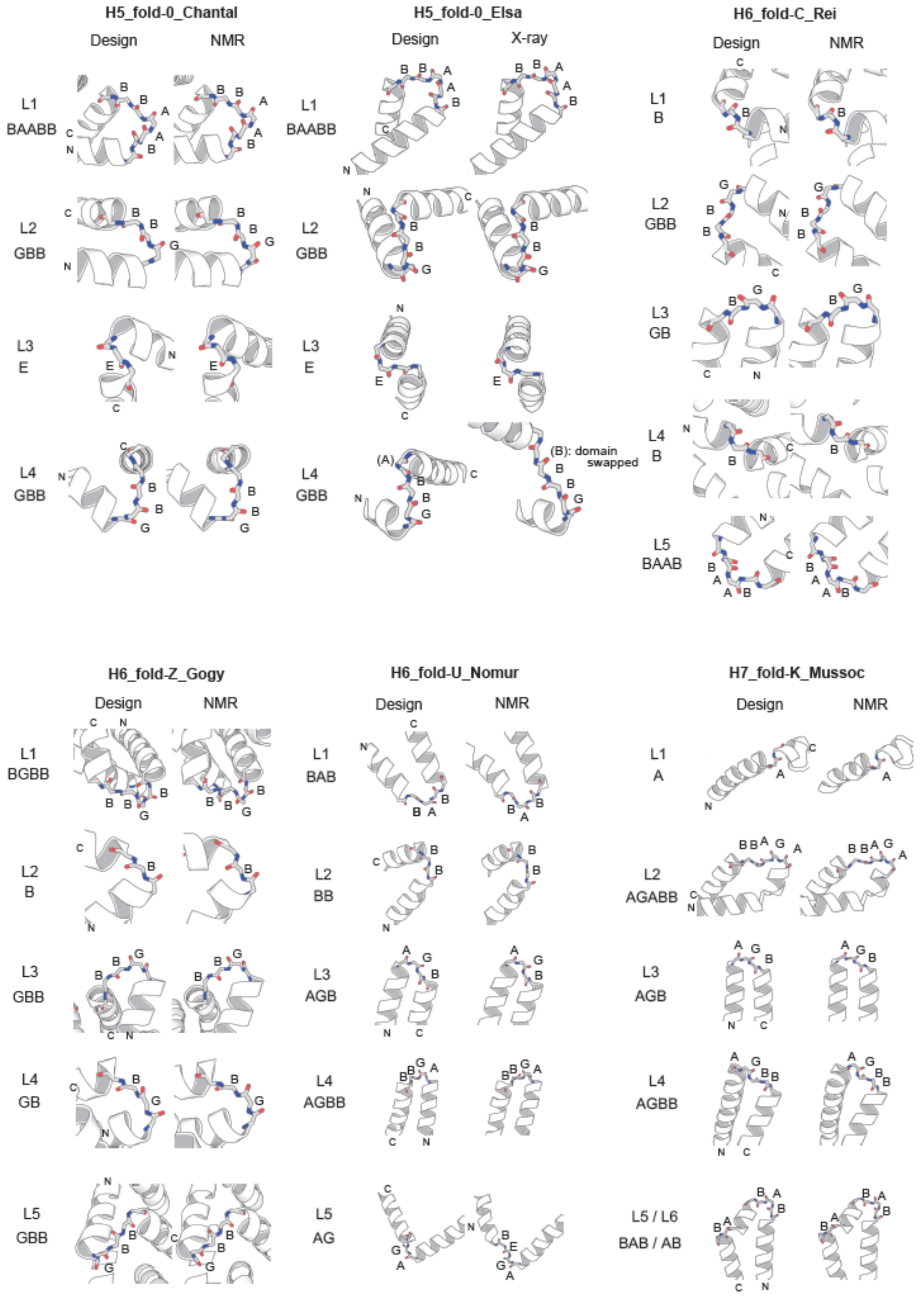

Supplementary Fig. 10 | Comparison of loop geometries of HLH motifs between design models and experimental structures.

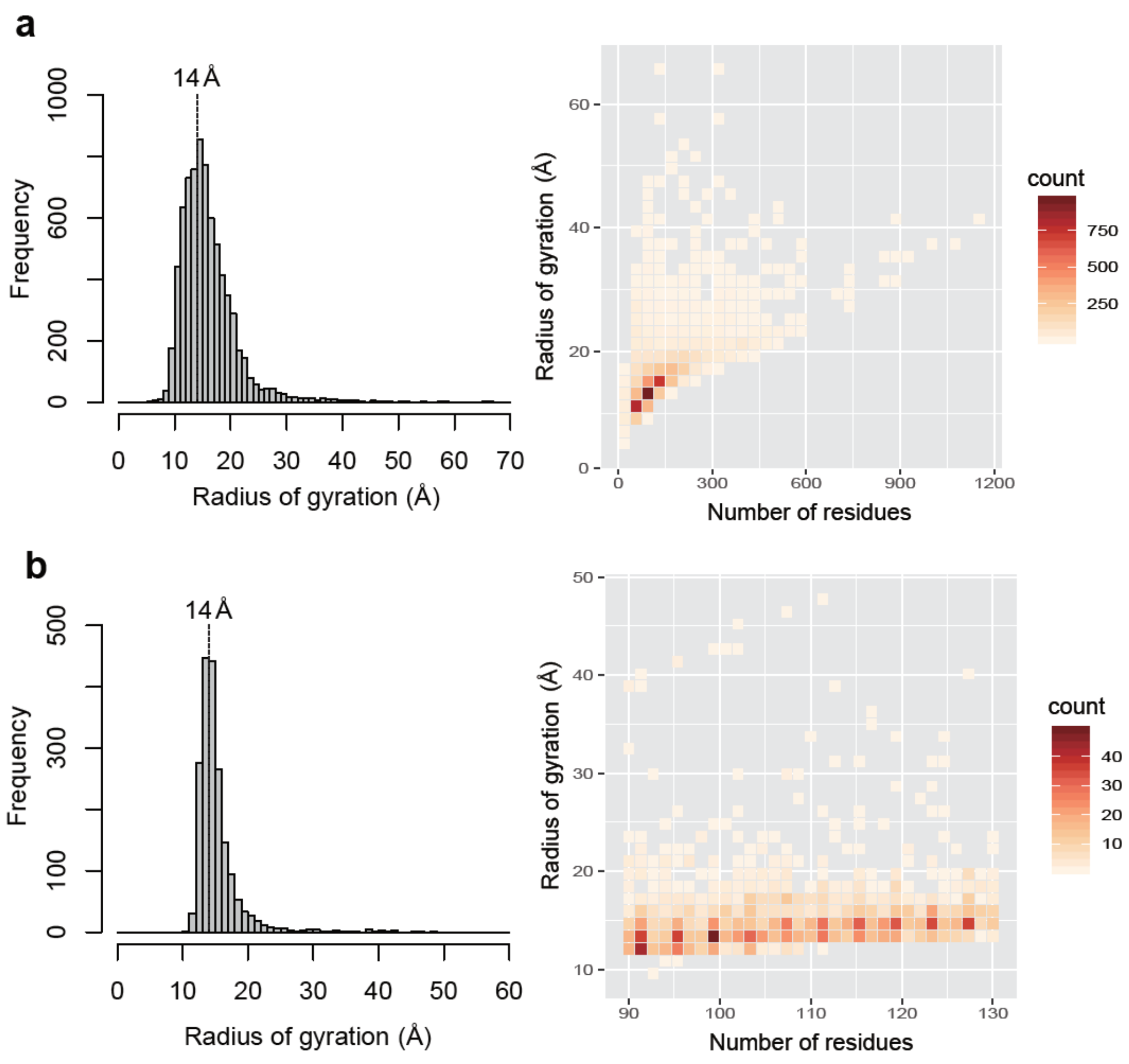

Supplementary Fig. 11 | Distribution of radius of gyration for naturally occurring all-α proteins.

a, The distribution of radius of gyration for naturally occurring all-α protein structures. **b**, The distribution of radius of gyration for naturally occurring all-α protein structures with 90 to 130 residues. These distributions are calculated using the proteins found in the mainly-α class in the CATH database[1] with sequence identity less than 40%.

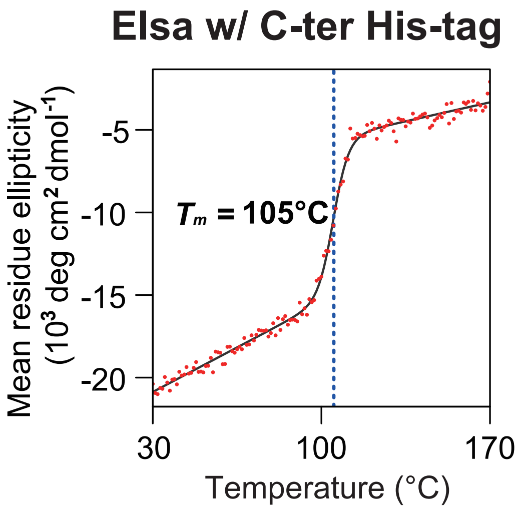

Supplementary Fig. 12 | Thermal denaturation of Elsa with the C-terminal His-tag.

Thermal denaturation was measured at 222 nm by CD under the pressure of 10 bar. The data were fit to a two-state model (solid line) to obtain the T_m.

H5_fold-0

ID	sequence	E-value
Ana	mGEEEKRLKEILELIARWYEHVREKDRTGTEEDIVRKAVEEAAKTHGTNPKEVLERIARA<br>IKKTGREQVARQAGTSEKTVEIIRRLWEREGslehhhhhh	0.70
Bill	mGEDEKELQRLLELLNRSYETEKRKDKSGSSKEWLRRAVEKAARQHGTSPSRLLELVQR<br>AIEKKGRKELAKRMGTSEEDVKLLEELSKEQGslehhhhhh	0.26
Chantal	mGEEEKEIDKLVELFAQAYEDAREKKRNGTPEEWVRDAIEEAARRVGRSRSRVVEALRR<br>YAEKHGKEELLKRAGITPEALKVIEKIEKEEGslehhhhhh	0.73
Chris	mGEEEKEIRKIVELFARAYKEAKEKKRNGTPEEWVRDAVEKAAQKVGRSRKDVVEALQK<br>FADQEGEEELAKQLGISPEALKVIKKIRKEEGslehhhhhh	0.65
Danny	mGEEEKEIERLVELFAQAFREVKEKDQTGTPEEIARKAVERAAREEGRSRKRVVEALENY<br>ARKKGEEELLKRVGMTPEVWKVVQQIKKEEGslehhhhhh	0.78
Elsa	mGEEQKEIETLVELFAEAFREAKRQKKNGTPEEWARDAVEEAARQQGRSRKDVVEALT<br>KYAQEQGRDELLKRLGITPEIYKVIQQIRKEEGslehhhhhh	0.093
Fay	mGEEQKELEKVLELFAQAFEQAKREKKNGTPEEWARDAVERAAQRVGRSRKDVVDLIE<br>KAAREQGEKKLLERLGISPEVLKVIKKIQKEEGslehhhhhh	0.46
Flanklin	mGEEEKEREELIRLFAKAFEEYKRKHKNGSKEEIARRALERAARERGRSRSRVVEALERE<br>ARQTGEDELLKQVGMDPEVWKVVQQIKKEEGslehhhhhh	1.6
Hermine	mGDEDEKKKRILEALLRAYEEWRKKDKTGSQEDVVRKAVERAAREAGTNPRDVLRVVQ<br>EEIEKTGPDELAKKIGTSPSVVELLRKVYEEEGslehhhhhh	0.91
Larry	mGEREKKIKEVVRRFAKAYEEVRKTNQNGSEKEIVERAVREVAKEEGTSPREVLKILVEFI<br>KRKGPEEFAKEAGSSSEAAKIIEELLRREGswslehhhhhh	2.0

	Ana	Bill	Chantal	Chris	Danny	Elsa	Fay	Franklin	Hermine	Larry
Ana	1	0.444	0.4	0.411	0.378	0.3	0.333	0.311	0.489	0.444
Bill	0.633	1	0.444	0.411	0.422	0.4	0.4	0.422	0.467	0.367
Chantal	0.578	0.656	1	0.722	0.656	0.667	0.611	0.567	0.344	0.4
Chris	0.589	0.611	0.822	1	0.611	0.678	0.667	0.544	0.378	0.4
Danny	0.556	0.6	0.756	0.733	1	0.689	0.589	0.733	0.378	0.378
Elsa	0.5	0.556	0.767	0.756	0.767	1	0.689	0.611	0.311	0.322
Fay	0.511	0.578	0.744	0.767	0.7	0.767	1	0.556	0.322	0.278
Franklin	0.544	0.578	0.7	0.711	0.789	0.733	0.678	1	0.367	0.367
Hermine	0.7	0.644	0.556	0.611	0.589	0.511	0.533	0.589	1	0.367
Larry	0.589	0.544	0.544	0.522	0.556	0.489	0.444	0.522	0.578	1

H6_fold-C

ID	sequence	E-value
Cyrus	mGSSVERAAEEVFQIIQQSPEVFERLIESMKEWMKKQGSSPDELRKLEKDLKEARKRAEEQKRQGNEEEMRRIVQKVLKSPAFKQAVQLMEEQEPNNPEVKKLKEAMEEVENGslehhhhhh	0.28
Dan	mGDEAKKAAEEMIKELSKNPELMKRLIKEMEKWLKQKGVSPDELERTRQEMETRIKEAIERKKRGDEESAREVMEEVLKSPAFKQAARNLEEQEPNNPEAQKMRELAERAEKGslehhhhhh	418
Max	mGEEAKKAAEEVLQLMDKSPDLMQQVIEEMKKVWKNKGASPEQLEKQERKVKTLVEEARKRKEEGNEEDARKVMLEVLKSPAFKQAVKRVEEQEPNNPEAKRLLELAERASQGslehhhhhh	91
Melvin	mGDDAKKMARKVLEIVTKSPDVMKTLIKRAEERLEKEGRSDEDKQRWRETIKKKVQKAIELQKEGNEEKAEETMEEVLKSPAFKQAVEMLEEQEPNNPEAQTLRRAMEEAERGslehhhhhh	1.8
Moca	mGDDAKKRARDLARTIAESPEVAERLAQALREKLENQGQSEDEIRSRVTELRKRLEEAIRQWRTGNKDEAMRVALEVLKSPAFKQAVEALEEQEPNNPEARKLLQMAEDVEKGslehhhhhh	0.047
Paul	mGQEARDAAREMLKEVERSPSVMKTVVEMARERAREEGQSPDQLKEIAREIEENVRRALELWRRGNSEKAEKVMEKVLKSPAFKQAMEMLEEQEPNNPEMNKVIELAKRASRGslehhhhhh	0.27
Rei	mGDEAEKQAERALELVRKSPDLLKKLLEAMAEELKRQGKSPDEIQKAKDEVKTKVEQAIREWKQGNEEQARKDMRKVLKSPAFKQAVKVMEEQEPNNPEVQELKKAMEEAERGslehhhhhh	2.4

	Cyrus	Dan	Max	Melvin	Moca	Paul	Rei
Cyrus	1	0.455	0.473	0.42	0.455	0.402	0.509
Dan	0.58	1	0.571	0.518	0.455	0.491	0.491
Max	0.616	0.705	1	0.464	0.402	0.473	0.527
Melvin	0.545	0.643	0.545	1	0.455	0.5	0.545
Moca	0.58	0.589	0.607	0.589	1	0.402	0.446
Paul	0.58	0.634	0.616	0.616	0.58	1	0.429
Rei	0.652	0.643	0.607	0.705	0.589	0.571	1

H6_fold-Z

ID	sequence	E-value
Gogy	mGDERKLEEVTEEMRKMAENMDGQDPEKVKEIVRRALQQMANDNPEVSEQLRELAKRKGTSPSEVIKDLAEQVWRAMERAREGDKDTARELIRKFADDLGISPEQVKKFIKIMREVQRKEDGslehhhhhh	0.065
Hoto	mGDDDEIRRILELLEKIARSLDGASEDTIREVIEQAIRDLQKQNPEFKKQVERVAKEQGTSPDEVIKKIVEIIWEAAERVRRGNKDEAEKVIRKLEKELGISPSTVRQLMEEIVRVLKEKKGslehhhhhh	0.0003
Kome	mGTDEELKKVDEELRRIQTQLDGQTEDEIRRIMEKVMKQLTKTNPEFKKQVKRVAEEQGTSPSEVLKEIIERLEKAAQEAKKGNTEEAQKIVKELAKTLGVTWSEIRRILEQIREEIERTQGslehhhhhh	0.35
Nazu	mGDDQELEEIMRLAEDVSREVKGQSPDELKKMMKKLLEELTKENPEFKETIRENAEKEGTSPSEVWRKVIERVEQIMRDVTKGDKEQARKEIEELRKELGVSLKDIKELMKRLKEVVKKSRGslehhhhhh	0.002
Seri	mGESDEIKKILQELEKIWQNIKGVSDEQIQEVFKQLVKELTREDPQFEKSVRRLAKEEGTSPSNVQEKLLKLIKDAAENVRRGNKDEAEKNIREIAKNLGVSHSDVLRIVKTLRELLEQAKGslehhhhhh	0.015
Siro	mGSSEDARTVVELLQKIRQEIEGLSPDQIKEVVKRAIDELEKENPQVSKNLEEKAQKQGTSPSKLKEKVAELIREAAERAQNGDSEEAKKLVEKIARILGISWKDVVRMMKEIIRRLRDENGslehhhhhh	0.018
Suzu	mGSKETLKKILKTLQEAKKELKGTSPEEIKKRIVKLLKEVAKESPEIKKNIKEEAERQGTSPSEVLKEIADLIREAMSRAEEGNSEEAEKMIDEIARIMGISWDQVLEILDRLVEQLKKDRGslehhhhhh	0.054

	Gogy	Hoto	Kome	Nazu	Seri	Siro	Suzu
Gogy	1	0.364	0.289	0.347	0.306	0.364	0.322
Hoto	0.554	1	0.446	0.364	0.471	0.421	0.355
Kome	0.57	0.661	1	0.372	0.372	0.314	0.397
Nazu	0.545	0.554	0.595	1	0.347	0.331	0.397
Seri	0.463	0.645	0.62	0.554	1	0.397	0.38
Siro	0.545	0.612	0.554	0.529	0.579	1	0.413
Suzu	0.529	0.57	0.62	0.595	0.521	0.636	1

H6_fold-U

ID	sequence	E-value
Cacyd	mGETDIRAALTALKTIIKKASNPEVAEAAKRLIEALKKASPEQRERAARKLIKALKALAKG DTERAQKLIKEAAEEAGLSPEDIKKLQKAARWLKEQGLVREALQAAQDVQDKGslehhhh hh	0.05
Hatake	mGETDIRAAQEALKVIIKKASNPEVAEAAKSLLEALKKASPEQRRRAAEKIIKALKALAKG DTERAVELLREAAEEAGLSPREIEKLRKAARWLKEQGLVREALRRAQEVQDNGslehhhh hh	0.72
Kazi	mGETERRAAERALEVLIRRATDPELQQAVKDLRKVLNTASPERQKRAAEKIFRALEAAA QGNKEKAKKLAEEAARVAGASPEIIRRLQKAVEKAQKRGIVKEARKAAQEVKENGswsleh hhhhh	0.041
Morit	mGDREIKAALTALKVIIKKASNPEVAEAAERLLRALKKASPEQRRRAAENIIRALKALAKG DTERAAKLLKKAAEEAGLSPDTIKKLKKAARWLKEQGLVREALKSAKEVTKKGslehhhh hh	0.004
Nomur	mGETKAKAAQEALRAAREQATTPEAQKALEELEKVLKTASPEQWRQAAEKIFEAFREAS NGNTEKAKKLLEEAARTAGASPEIIKKLASALERLAEEGAAKEAARQAEEVRKRGslehhh hhh	0.35
Sait	mGDKEAQAAQEAIKTAIKSATNPEAQRALKEFSRVANEASPDQWRKAADLIRKALEEAS RGNTERAERLARKAAKVVGASPEIIERLARAIRELARTGAAKKARQVAEKVKKKGslehhh hhh	0.051
Sho	mGEEVLKAAKRALEVIIRRATDPELQQAVKELQKILSTASDERRERAAKEIYRAAEAAAQ GNKEEARKRLEKAAKELGASPEQIEKAKKALDEAQKRGIVKEAKEEAEKQQRKGswsleh hhhhh	0.15
Takin	mGDTEAKAASRALQTIIDQATDPELKKALEDLRRRAEEASPDQWRQIAKAILKALELLDR GNTEEARKELEKAARKAGASPEVAKKLQKAFSEATKQGIAKEASKEAQKVRDKGslehhh hhh	0.47

	Cacyd	Hatake	Kazi	Morit	Nomur	Sait	Sho	Takin
Cacyd	1	0.823	0.487	0.779	0.442	0.416	0.434	0.451
Hatake	0.876	1	0.496	0.779	0.487	0.434	0.434	0.425
Kazi	0.619	0.619	1	0.46	0.549	0.46	0.628	0.513
Morit	0.858	0.841	0.593	1	0.469	0.434	0.451	0.478
Nomu	0.531	0.54	0.646	0.558	1	0.54	0.451	0.513
Sait	0.531	0.531	0.611	0.558	0.664	1	0.407	0.504
Sho	0.54	0.522	0.726	0.549	0.531	0.558	1	0.504
Takin	0.566	0.522	0.637	0.558	0.593	0.593	0.566	1

H7_fold-K

ID	sequence	E-value
Chario	mGDEEREKLRKIARKALKDAASEAKKRGIITPEAIERIANLLADAAQEWKEGNEERAEKL LRKAAKVFVEAAKKTGASADEARRVLERIRKALSNAPSDELRAIAKSPRLKKAIKEAIEEI RRIEEETGslehhhhhh	0.49
Dark	mGDDERKKIKEAAREAVERALEDAKRQGIVSPESLERIAEKIARAAEALEKGNSEKARKLI EEAARTILKEAERAGASSRWVEEILERLRQALEKAPSPRLQKLAQSPVFKTALEKIIREAR KREKKRGslehhhhhh	0.002
Jazzbird	mGDDDRKKLEQTVEEAIRKALEDAVRSGAITPEAAERIAQEIAEAAAQWRQGNTEEAEK AARRAIKVLLEAARKSGASSDQISELLERIREALSNAPSPEVQQLAKSPRFQQVLKEAKKE AKRIEKETGswslehhhhhh	0.000002
Mussoc	mGDEDKEKLKREAERALSEALSEFEKQGKITPETLKRLAEEIAEAALAQQQGDSERLEKA ARRFAETLLRALKESGASAEEIEEAIERIRKALSKAPSPQLQKLANSPQWQTALQEAIKKA RQEKKEKGslehhhhhh	0.64
NewWave	mGGSDRSDELRKQAEEAARKAFEEAKKRGVITPDAIKKVAEEIARAAELWRQGNTEEAE KAVRNAIKVILEAAKKSGASSQEVSDALERLRQALENAPSPEVQQLAKSPRFQQVIEEAEK EAKRIEKETGslehhhhhh	0.043
R3	mGDDDREKLRETAREALREALSKFEKQGKITPEVLERLAEAIAEAASALEKGDSERLEKA ARKFAQILLESLKKSGASAEEIEEALRLAQKALSKAPSPQLQRVANSPRWQEALNEAIKRA REEEKRTGslehhhhhh	0.32
Rush	mGEDEEKKIEEAARQAVETALEDARRQGQITPERIEKIAEKIARAATALRRGNKEEAIKLI KEAAQIIVEAARESGASSEWIEEVLRRIEEALRRAPSPQLQKLADSPAFQKAAREAIERAR EEEERTGswslehhhhhh	0.079
Third	mGDDEAKKIEDAAREAVKKALSEALKRGTITPDVIEEIAQRLAEAAIALEEGNKEKAAKL AKEALAKLIEEAAREGASPRWIERLIDRIEKQLANAPSPQLQKLAESPVFKKVLEEAKQRA DEVRRKTGslehhhhhh	0.028

	Chario	Dark	Jazzbird	Mussoc	NewWave	R3	Rush	Third
Chario	1	0.422	0.531	0.461	0.531	0.484	0.461	0.453
Dark	0.578	1	0.469	0.453	0.453	0.477	0.594	0.516
Jazzbird	0.68	0.602	1	0.508	0.688	0.516	0.516	0.477
Mussoc	0.594	0.617	0.609	1	0.453	0.711	0.453	0.406
NewWave	0.656	0.625	0.773	0.594	1	0.445	0.453	0.406
R3	0.594	0.617	0.609	0.781	0.562	1	0.523	0.445
Rush	0.602	0.695	0.625	0.594	0.578	0.664	1	0.555
Third	0.578	0.656	0.617	0.539	0.539	0.594	0.656	1

Supplementary Table 1 | Designed sequences and sequence identity among them.

Computationally designed sequences are shown in uppercase and residues added to allow expression, purification, and the spacer between the designed sequence and the C-terminal His-tag are shown in lowercase. The rightmost column shows PSI-BLAST E-values against the nr database of nonredundant protein sequences.

	**Expressed**	**Soluble**	**α-protein CD spectrum (20 °C)**	**Monomeric**	**Well-resolved HSQC**
**Ana**	Y	N			
**Bill**	Y	Y	Y	Y	Y
**Chantal**	Y	Y	Y	Y	Y
**Chris**	Y	Y	Y	N	
**Danny**	Y	Y	Y	N	
**Elsa**	Y	Y	Y	Y	Y
**Fay**	Y	Y	Y	Y	†
**Franklin**	Y	Y	Y	Y	Y
**Hermine**	Y	Y	Y	Y	Y
**Larry**	Y	Y	Y	Y	Y

Supplementary Table 2 | Summary of experimental results of 10 designs for H5_fold-0.

Each row corresponds to the results for each design. The columns give the results for each experimental test, of which the details are described in Extended Data Table 1. Each test was performed sequentially from the left to the right; well-behaved designs at one test (Y) go to the next one and not well-behaved designs (N) end the tests.

† The experiment was not conducted due to low concentration.

	Expressed	Soluble	α-protein CD spectrum (20 °C)	Monomeric	Well-resolved HSQC
**Cyrus**	Y	Y	Y	Y	Y
**Dan**	Y	Y	Y	Y	Y
**Max**	Y	Y	Y	Y	‡
**Melvin**	Y	N			
**Moca**	Y	Y	Y	Y	†
**Paul**	Y	Y	Y	N	
**Rei**	Y	Y	Y	Y	Y

Supplementary Table 3 | Summary of experimental results of 7 designs for H6_fold-C.

The summary was given in the same way as Supplementary Table 2.

‡ The experiment was not conducted due to high nucleotide concentration.

† The experiment was not conducted due to low concentration.

	**Expressed**	**Soluble**	**α-protein CD spectrum (20 °C)**	**Monomeric**	**Well-resolved HSQC**
**Gogy**	Y	Y	Y	Y	Y
**Hoto**	Y	Y	Y	N	
**Kome**	Y	Y	Y	N	
**Nazu**	Y	Y	Y	Y	Y
**Seri**	Y	Y	Y	Y	Y
**Siro**	Y	Y	Y	Y	Y
**Suzu**	Y	Y	Y	N	

Supplementary Table 4 | Summary of experimental results of 7 designs for H6_fold-Z.

The summary was given in the same way as Supplementary Table 2.

	Expressed	Soluble	α-protein CD spectrum (20 °C)	Monomeric	Well-resolved HSQC
**Cacyd**	Y	Y	Y	Y	‡
**Hatake**	Y	Y	Y	Y	Y
**Kazi**	Y	N			
**Morit**	Y	N			
**Nomur**	Y	Y	Y	Y	Y
**Sait**	Y	N			
**Sho**	Y	Y	Y	Y	Y
**Takin**	Y	Y	Y	Y	Y

Supplementary Table 5 | Summary of experimental results of 8 designs for H6_fold-U.

The summary was given in the same way as Supplementary Table 2.

‡ The experiment was not conducted due to high nucleotide concentration.

	Expressed	Soluble	α-protein CD spectrum (20 °C)	Monomeric	Well-resolved HSQC
**Chario**	Y	N			
**Dark**	Y	Y	Y	N	
**Jazzbird**	Y	Y	Y	Y	Y
**Mussoc**	Y	Y	Y	Y	Y
**NewWave**	Y	Y	Y	Y	Y
**R3**	Y	Y	Y	Y	Y
**Rush**	Y	Y	Y	Y	Y
**Third**	Y	Y	Y	Y	Y

Supplementary Table 6 | Summary of experimental results of 8 designs for H7_fold-K.

The summary was given in the same way as Supplementary Table 2.

	^1H_N, 15Nα	^1H_N, 15Nα, $^{13}C\beta$, $^{13}C\alpha$, $^{13}C'$	All atoms
H5_fold-0_Chantal	172 / 174 (98.9 %)	426 / 433 (98.3 %)	897 / 918 (97.7 %)
H6_fold-C_Rei	214 / 216 (99.1 %)	540 / 547 (98.7 %)	1165 / 1201 (97.0 %)
H6_fold-Z_Gogy	234 / 236 (99.2 %)	585 / 587 (99.7 %)	1280 / 1288 (99.4 %)
H6_fold-U_Nomur	217 / 222 (97.7 %)	551 / 560 (98.4 %)	1101 / 1131 (97.3 %)
H7_fold-K_Mussoc	229 / 242 (94.6 %)	583 / 611 (95.4 %)	1156 / 1210 (95.5 %)

Supplementary Table 7 | Completeness of assigned atoms in NMR structure determination

The first residue at the N-terminus, 6xHis-tag, the one residue immediately before the His-tag at the C-terminus, and the residues classified as "Dynamic" or "Warning" by TALOS+ dihedral angle prediction[2], respectively based on order parameter prediction[3] and statistic clustering of triplet search algorithm, are not included for the calculations: 1, 91-99 for H5_fold-0_Chantal; 1, 115-121 for H6_fold-C_Rei; 1, 124-130 for H6_fold-Z_Gogy; 1, 116-122 for H6_fold-U_Nomur; 1, 127-137 for H7_fold-K_Mussoc.

	RMSD between design and NMR (Å)		T_m (°C)
	**Cα atoms**	**Heavy atoms**	
**H5_fold-0_Chantal**	1.9	3.8	118
**H5_fold-0_Elsa**	domain-swapped		106
**H6_fold-C_Rei**	1.5	3.6	105
**H6_fold-Z_Gogy**	1.7	3.6	122
**H6_fold-U_Nomur**	3.1	4.4	116
**H7_fold-K_Mussoc**	2.2	4.0	139

Supplementary Table 8 | Summary of experimental results for the six designs of the five folds.

The second and third columns show the average RMSD between the design model and the 20 NMR structures using Cα atoms and heavy atoms respectively. The computationally designed regions were used for RMSD calculations. The last column shows the melting temperature T_m.

	L1	L2	L3	L4	L5	L6
**H5_fold-0_Chantal**	BAABB/BAABB	GBB/GBB	E/E	GBB/GBB	-	-
**H5_fold-0_Elsa**	BAABB/BAABB	GBB/GBB	E/E	GBB/GBB	-	-
**H6_fold-C_Rei**	B/B	GBB/GBB	GB/GB	B/B	BAAB/BAAB	-
**H6_fold-Z_Gogy**	BGBB/BGBB	B/B	GBB/GBB	GB/GB	GBB/GBB	-
**H6_fold-U_Nomur**	BAB/BAB	BB/BB	AGB/AGB	AGBB/AGBB	AG/AGEB	-
**H7_fold-K_Mussoc**	A/A	AGABB/AGABB	AGB/AGB	AGBB/AGBB	BAB/BAB	AB/AB

Supplementary Table 9 | Comparison of ABEGO-based loop geometries of HLH motifs between design models and experimental structures for the six designs of the five folds.

For the five designs except H5_fold-0_Elsa, the loop types for the design model (left) and the most frequent loop type in the NMR conformers (right) are shown. For H5_fold-0_Elsa, the loop types for the design model (left) and those of the crystal structure (right) are shown. The crystal structure of H5_fold-0_Elsa was domain-swapped due to the torsion angle difference of the residue immediately after the L4, Pro76, from A to B in the ABEGO representation.

References

1. Orengo, C.A. et al. CATH--a hierarchic classification of protein domain structures. *Structure* **5**, 1093-1108 (1997).
2. Shen, Y., Delaglio, F., Cornilescu, G. & Bax, A. TALOS+: a hybrid method for predicting protein backbone torsion angles from NMR chemical shifts. *J Biomol NMR* **44**, 213-23 (2009).
3. Berjanskii, M. & Wishart, D.S. NMR: prediction of protein flexibility. *Nat Protoc* **1**, 683-8 (2006).
